# Decoding cognitive aging: how white matter tracts and demographics distinguish potential Super-Agers

**DOI:** 10.1007/s11357-025-01566-0

**Published:** 2025-02-26

**Authors:** Mohammad Fili, Parvin Mohammadiarvejeh, Guiping Hu, Auriel A. Willette

**Affiliations:** 1 School of Industrial Engineering and Management, Oklahoma State University, Stillwater, OK 74078 USA; 2 Institute for Health Computing, University of Maryland Medical System, Linthicum, MD 21090 USA; 3 Department of Neurology, Rutgers University, New Brunswick, NJ 08901 USA

**Keywords:** Cognitive classification, Cognitive scoring, Diffusion magnetic resonance imaging (dMRI), Positive-Agers, Super-Agers, UK Biobank

## Abstract

Most adults experience age-related cognitive decline. However, “Positive-Agers” exhibit superior cognition compared to their age-matched peers. Distinguishing between those with superior cognitive performance and those with cognitive decline over time could better inform treatment therapies in older adults. We developed an algorithm called *Opti*mal *C*ognitive *S*coring (OptiCS) that accurately differentiates “Positive-Agers” from “Cognitive Decliners.” This study draws on a cohort of 5797 participants longitudinally enrolled in the UK Biobank. Using a predictive pipeline, OptiCS could strongly differentiate Positive-Agers versus Cognitive Decliners (area under the curve, or AUC of 83%). The top diffusion MRI attributes highlighted tracts implicated in pathological aging, including the fornix from the hippocampus, the tapetum from the splenium of the corpus callosum, and other key tracts. This study provides three key insights: (I) The proposed algorithm offers a robust cognitive scoring system for subtle cognitive changes, (II) OptiCS can use diffusion MRI to accurately gauge cognitive performance, and (III) OptiCS provides a predictive framework for early detection of cognitive decline.

**Supplementary Information:**

The online version contains supplementary material available at 10.1007/s11357-025-01566-0.

## Introduction

Cognitive aging is a natural lifelong phenomenon in which levels of performance in many cognitive tasks gradually decrease [1, 2]. However, prior research has uncovered inconsistent patterns of age-related changes in various cognitive domains among healthy adults. While some individuals experience a constant and rapid decline in cognition, others may exhibit a slight decline, maintain stable cognitive function, or even demonstrate improvement over their lifetime [3]. Moreover, there exist individuals in their 80s or older, called Super-Agers, whose cognitive abilities perform at least as well as those of middle-aged adults [4]. Similarly, subgroups of participants in the UK Biobank study show different patterns in their cognitive trajectories relating to verbal and numerical reasoning [5, 6]. Among these largely middle-aged adults, identifying neurological differences associated with aging, between patterns of cognitive gain and loss, can offer valuable insights toward promoting successful aging among older adults. In contrast, older adults with mild cognitive impairment (MCI) or Alzheimer’s disease (AD) have cognitive decline due to biological changes. Their risk and cognitive profiles are thus much more difficult to track due to the complex nature of pathological aging [7, 8].

Diffusion magnetic resonance imaging (dMRI) is an imaging technique that quantifies the Brownian diffusion of water within the tissue environment. In the brain, this technique provides detailed information about parenchymal tissue integrity by monitoring water movement around dense and less dense obstacles [9, 10]. These obstacles include cell membranes and organelles for gray matter, fatty myelin sheaths for white matter, and water-like cerebrospinal fluid. For instance, one study found reduced fractional anisotropy (FA) and increased mean diffusivity (MD) in older versus middle-aged adults in the centrum semiovale (i.e., deep white matter) and whole brain [11]. Schiavone et al. [12] reported that less FA was associated with white matter atrophy because of age-related decline in multiple cognitive domains, such as episodic memory, executive function, working memory, and information processing speed. Similarly, changes in the anterior corpus callosum or posterior cingulate white matter integrity may be related to processing speed and AD-like changes [13, 14]. Likewise, increased diffusivity in cholinergic tracts may reflect poorer executive function performance [15]. In concert, among participants with MCI, there is increased axial diffusivity (AxD) and radial diffusivity (RD) parahippocampal cingulum [16]. Finally, among Super-Agers compared to normal aging controls, there was more microstructural integrity in the corpus callosum and superior longitudinal fasciculus [17].

To date, most studies have evaluated the association between dMRI values in various brain tracts or regions and cognitive performance, usually in normally aging adults. Yet, no large-scale study has investigated a comprehensive set of dMRI attributes to identify “Positive-Agers,” who are middle-aged to aged adults with superior cognitive performance, compared to adults showing age-related cognitive decline (“Cognitive Decliners”). The key distinction between Positive-Agers and Super-Agers is age: Super-Agers are 80 years or older, whereas Positive-Agers are younger. In our study, Positive-Agers were between 55 and 70 years old at the baseline visit.

Several studies have proposed models or frameworks to identify cognitive classes using other data sources. For instance, Parvin et al. developed predictive models to distinguish between adults experiencing cognitive decline and those with cognitive gains over time using blood biochemistry and immunology. Their study demonstrated that bioenergetic and vascular factors in middle age can effectively predict cognitive decline with high accuracy [6]. Another study utilized multiple cognitive assessments to identify middle-aged adults with declining and improving cognitive trajectories over time, subsequently developing classification models based on structural brain magnetic resonance imaging (sMRI). This study highlighted key brain regions that play a crucial role in distinguishing these cognitive groups [18]. Fili et al. proposed a hybrid algorithm combining machine learning and optimization techniques to differentiate cognitive decline from positive aging using resting-state functional magnetic resonance imaging (rsfMRI) features. Their study introduced a mathematical definition for these two groups based on cognitive test results [19]. Furthermore, other studies have explored classification approaches using rsfMRI and lifestyle data, including one that applied multiple predictive models to classify Super-Agers and natural cognitive decliners in adults over 80 years old [20], and another that employed logistic regression to distinguish cognitive decliners from those who maintained cognitive function based on lifestyle factors [21].

Here, we propose a powerful algorithm that separates cognitive classes (i.e., “Positive-Ager” from “Cognitive Decliner”) according to individuals’ cognitive scores obtained from a standardized set of cognitive tests. The optimal cognitive classes are then employed to quantify the importance of features (i.e., dMRI attributes and demographic characteristics) in correctly identifying participants as Positive-Agers or Cognitive Decliners. The proposed algorithm operates as an iterative process, integrating optimization and machine learning (ML) techniques. The ML component employs a regularized logistic regression model as a predictive tool to identify the relationship between input features and cognitive classes. Meanwhile, the optimization component ensures that the algorithm’s settings (i.e., cognitive scoring parameters and model’s hyperparameters) are fine-tuned to achieve optimal performance. Detailed descriptions of the proposed algorithm can be found in the “Classification procedure” section.

## Materials and methods

This study involved 5797 participants who were longitudinally enrolled in the UK Biobank cohort. The UK Biobank is an extensive, long-term biomedical research initiative that has gathered data from over 500,000 adults in the UK. This data collection was originally conducted at baseline and included questionnaires, physical assessments, cognitive tests, imaging, and biological samples [22]. For this analysis, we focused on participants who were at least 55 years old at their initial visit between 2006 and 2010 (${t}_{1}$). A subgroup of these participants was followed up in 2012–2013 for a second visit (${t}_{2}$), during which they provided informed consent and completed a touchscreen questionnaire, a verbal interview, and measures of eye health and body size, alongside blood and urine sample collections. Subsequent follow-ups in 2014 (${t}_{3}$) and 2019 (${t}_{4}$) involved detailed imaging of the brain, heart, and other body parts [23, 24]. Due to concerns about maintaining a sufficient sample size, we limited our analysis to the ${t}_{3}$ brain imaging data. Including the ${t}_{4}$ data would have reduced our sample size from 5797 to 1416 observations.

Throughout the study, socio-demographic characteristics, occupation, lifestyle, and cognitive function were collected via touchscreen or laptop-administered questionnaires. The UK Biobank protocol received approval from the Northwest Multi-Centre Research Ethics Committee.

### Data

We used three data sources: (1) cognitive tests, (2) dMRI data, and (3) demographic information. Cognitive tests were the primary input for computing cognitive scores and determining who belongs to the “Positive-Ager” vs. “Cognitive Decliner” class. Subsequently, dMRI and demographic data were used to classify participants’ cognitive classes.

#### Cognitive tests

At three distinct time points, ${t}_{1}$, ${t}_{2}$, and ${t}_{3}$, a series of cognitive tests were administered, including assessments of fluid intelligence (FI), pairs matching memory (PMM), and reaction time (RT). FI is assessed through tasks that measure verbal and numerical reasoning, requiring participants to solve problems based on logic rather than prior knowledge. In this test, participants were presented with 13 multiple-choice questions encompassing logical, numerical, and combinatorial challenges. The performance was quantified by the number of correct responses given within a 2-min time frame [25]. The PMM task evaluated visual memory. Participants viewed a screen displaying several pairs of matching cards during this test. They were instructed to memorize the positions of these pairs. Afterward, the cards were turned face down, and participants were tasked with identifying the matching pairs with the fewest possible attempts. The performance was scored based on the number of errors made during their second trial [26]. Finally, the RT test aimed to measure processing speed through a 12-round card game. In this test, participants were shown pairs of cards with symbols. They were instructed to press a button on the desk as quickly as possible if the symbols matched. They were to refrain from pressing the button if the symbols did not match. The first five rounds served as practice trials and were not included in the final score. The score was calculated as the average reaction time for pressing the button across the four rounds where the symbols matched [27].

For our analysis, we utilized cognitive test data from time points ${t}_{1}$ and ${t}_{3}$. The decision to exclude ${t}_{2}$ was primarily driven by logistical considerations. Incorporating data from ${t}_{2}$ alongside ${t}_{1}$ and ${t}_{3}$ would have significantly reduced our sample size from 5797 participants to only 911. Thus, to maintain a robust sample size, we focused on the data from ${t}_{1}$ and ${t}_{3}$.

Since the tests were skewed, the PMM and RT tests were transformed using $\text{log}\left(x+1\right)$ and $\text{log}\left(x\right)$, respectively.

#### dMRI data

UK Biobank conducted baseline neuroimaging in 2014 (${t}_{3}$) and a follow-up visit in 2019 (${t}_{4}$). Imaging assessments were done at three centers on the same Siemens Skyra scanners with a standard Siemens 32-channel head coil [28]. UK Biobank dMRI features included classic anisotropy measures like fractional anisotropy (FA), mean diffusivity (MD), L1 (axial diffusivity, or AxD), and the average of L2 and L3 (radial diffusivity, or RD). Additional metrics based on neurite orientation dispersion and density imaging (NODDI) included mode of anisotropy (MO), orientation dispersion (OD), intra-cellular volume fraction (ICVF), and isotropic or free water volume fraction (ISOVF) [28]. The tract values of the left and right hemispheres were averaged for each feature. In total, we had 135 dMRI features.

#### Demographic data

The demographics dataset comprised age, sex, body mass index (BMI), socioeconomic class, education level, skin color, handedness, waist circumference, and tobacco use and type. The age of each participant at the time of the initial visit was identified as their age in years. Socioeconomic classes were categorized based on UK Biobank coding into five strata according to the average total household income, provided in British pounds: under-class (< 18,000), lower-class (18,000–30,999), middle-class (31,000–51,999), upper-middle (52,000–100,000), and upper-class (> 100,000). The education level was categorized into four levels: “college or higher education,” “post-secondary or vocational education,” “secondary education,” and no education level from any of the previous groups. Lastly, tobacco smoking status was another categorical variable with three levels: “never smoked,” “previously smoked,” and “currently smoking.”

### Classification procedure

In this study, we developed an algorithm named *Opti*mal *C*ognitive *S*coring (OptiCS), designed to quantify cognitive performance and ensure that the resulting scores would have the highest possible correlation with the input features (i.e., dMRI and demographics). OptiCS integrates both machine learning and optimization techniques. The optimization component is tasked with determining the optimal parameters for cognitive scoring, while the machine learning component identifies and models the relationships between the input features and cognitive classes (i.e., “Positive-Agers” and “Cognitive Decliners”).

**Fig. 1:**
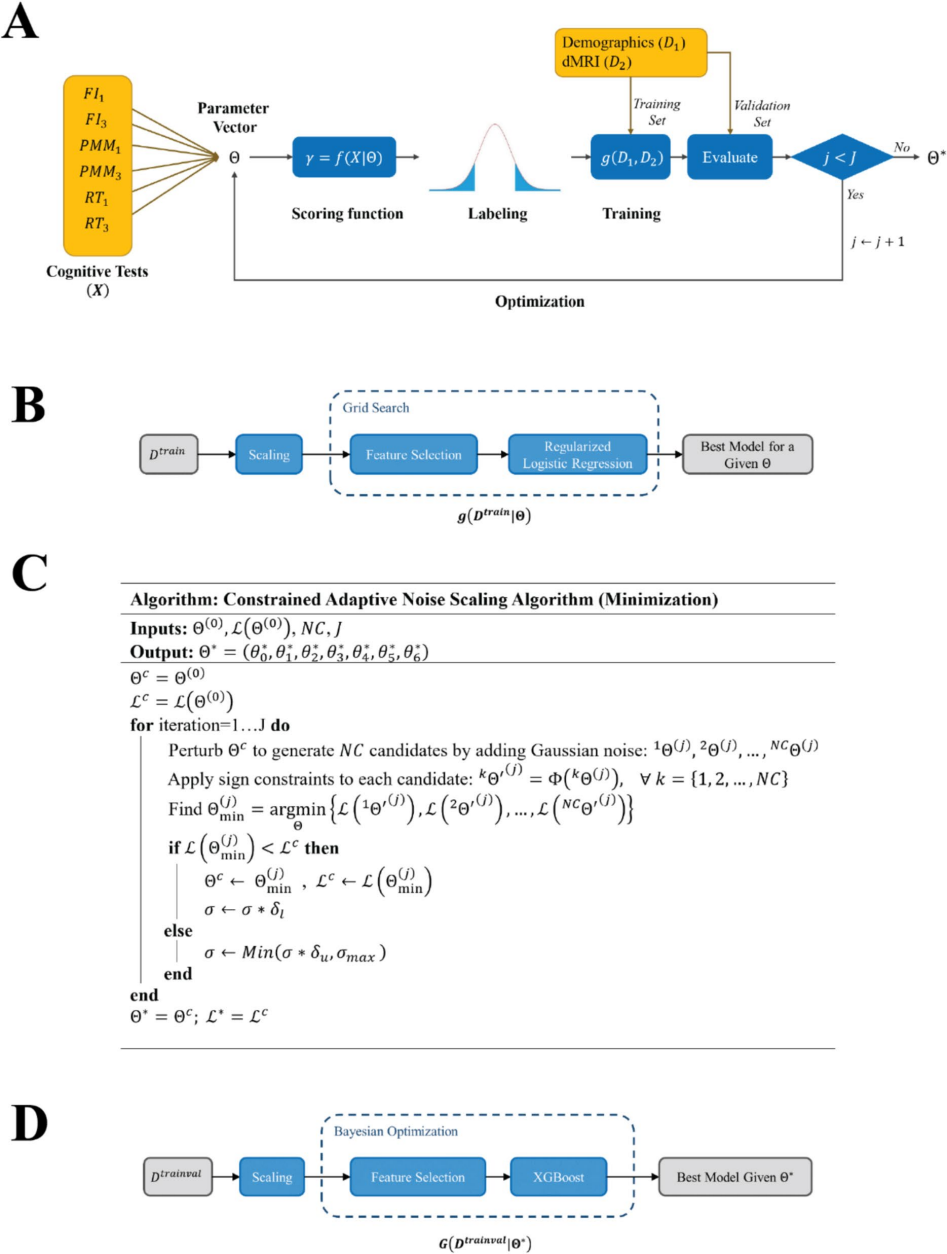
Schematic of different components of the algorithm. **A** Main flowchart of the algorithm. **B** The schematic view of the predictive pipeline $g$ responsible for predicting the class labels, given the input features and parameter vector $\Theta$ in each iteration. **C** Pseudocode of the CANS optimization algorithm, responsible for optimizing the parameter vector. **D** Schematic view of the final predictive pipeline $G$ applied on the optimal parameter vector ${\Theta }^{*}$

The OptiCS algorithm is comprised of five main components: (I) scoring, (II) labeling, (III) training, (IV) evaluation, and (V) optimization. The algorithm employs an iterative process based on a feedback loop for optimization. In each iteration, the algorithm modifies the parameters of a scoring function and assigns class labels according to the scores obtained. Subsequently, a regularized logistic regression is trained to predict these class labels using the dMRI and demographic input features. The performance of the model is then evaluated on a distinct validation set, which provides the basis for a loss function. This loss function is minimized across iterations to ensure the optimality of results (see Fig. 1A for the flowchart and Supplementary Fig. S1 for the pseudocode of the algorithm).

After finding the optimal parameters for the scoring function, we employed a more advanced predictive pipeline to classify the cognitive classes. At last, the performance of the model was evaluated on an untouched test set.

#### Cognitive scoring

Consider a mapping function $f$: ${\mathbb{R}}^{m}\to [0, 1]$ which takes $m$ cognitive tests as the input and computes a cognitive score $\gamma$ in the range between 0 and 1: 

1 $$\gamma =f\left(\text{X }|\Theta \right)$$

$X$ denoted cognitive tests which are standardized by removing the mean and scaling to unit variance before feeding into the scoring function. Standardized cognitive exam values are denoted by superscript $s$ in Eq. (2).

2 $$X={\left(1, F{I}_{1}^{s}, F{I}_{3}^{s}, PM{M}_{1}^{s}, PM{M}_{3}^{s}, R{T}_{1}^{s}, R{T}_{3}^{s}\right)}^{\prime}$$

3 $$\Theta =({\theta }_{0}, {\theta }_{1},{\theta }_{2}, {\theta }_{3}, {\theta }_{4},{\theta }_{5}, {\theta }_{6})$$

where $\Theta$ is the parameter vector comprising seven parameters with ${\theta }_{0}$ as the bias term, and ${\theta }_{1}$ to ${\theta }_{6}$ corresponding to the six cognitive exams ($m=6$), in the same order defined in $X$.

In this study, we used a logistic function for cognitive scoring:

4 $$\gamma =f\left(X \right|\Theta )=\upsigma \left(\Theta X\right)=\frac{1}{1+{e}^{-\Theta X}}$$

Given that the fluid intelligence score (*FI*) is known to be positively correlated with cognitive performance, while the *PMM* and *RT* scores are negatively correlated, we leveraged this prior knowledge by imposing constraints on the feasible domains for a parameter vector (see Supplementary Table S1 for details). These constraints ensured that higher cognitive scores were associated with a greater likelihood of an individual being classified as a “Positive-Ager.”

#### Labeling

Given the set of scores from the previous step, ${\Gamma }^{train}=\{{\gamma }_{1}, {\gamma }_{2}, \dots , {\gamma }_{N}\}$ for $N$ participants in the training dataset, we obtained the lower and upper bounds for thresholding as

5 $$lb= {\mathcal{Q}}_{q}\left({\Gamma }^{train}\right) , ub={\mathcal{Q}}_{1-q}\left({\Gamma }^{train}\right)$$

where ${\mathcal{Q}}_{q}({\Gamma }^{train})$ is the $q$-th quantile of training cognitive scores. Then, the labels, $y$, are assigned according to the Eq. (6) for all participants:

6 $$y_i=\left\{\begin{array}{c}`\!\ \!`Cognitive\;Decliner\!",if\gamma_i\leq lb\\`\!\ \!`Positive-Ager\!",if\gamma_i\geq ub\end{array}\right.,\forall i=\{1,2,\dots,N\}$$

According to this labeling function, participants with scores higher than the $q$-th upper quantile of cognitive scores are classified as “Positive-Agers,” whereas participants with scores below the $q$-th lower quantile of cognitive scores are “Cognitive Decliners.” Any participant with a score between the upper and lower thresholds is considered to belong to the “Normal-Aging” class, which was not of interest, for this report. The lower and upper thresholds found in the training phase, $lb$ and $ub$, were then applied to the validation and test sets.

In this study, we tested four quantile options: 0.10, 0.15, 0.20, and 0.25.

#### Training

We used logistic regression with L1 regularization as the classifier in the algorithm. A recursive feature elimination procedure was applied before training the main model to reduce the dimensionality of the feature space. For this purpose, we opted for the top 150 features ($\mathcal{F}=150$) according to the amount of impurity reduction in a decision tree model with a maximum depth (${m}_{fs}$) of 50. To achieve optimal performance, we incorporated hyperparameter tuning using a grid search approach with a focus on the regularization strength in the logistic regression model (see Fig. 1B for the schematic view of the predictive pipeline $g$). Given the parameter vector $\Theta$, the goal is to find the best logistic regression model that predicts the cognitive classes using the training data ${D}^{train}=[{D}_{1}^{train}, {D}_{2}^{train}]$, where ${D}_{1}$ and ${D}_{2}$ are referring to the demographic and dMRI data, respectively.

#### Evaluation

To evaluate the predictive pipeline $g$ in each iteration of the algorithm, we used the area under the ROC curve (AUC) metric in the objective function. For this purpose, we aimed to minimize the loss function in Eq. (7):

7 $$\mathcal{L}\left(\Theta \right)=1-AUC\left(g\left({D}^{val}|\Theta \right)\right)$$

where $g\left({D}^{val}|\Theta \right)$ is the predictive pipeline evaluated on the validation data ${D}^{val}=\left[{D}_{1}^{val}, {D}_{2}^{val}\right]$ given the parameter vector $\Theta$.

#### Optimization

The proposed algorithm uses a modified version of the adaptive noise scaling (ANS) method, named constrained adaptive noise scaling (CANS), designed to optimize the parameters of the scoring function. CANS is an iterative optimization algorithm that enhances the simulated annealing approach by dynamically adjusting the search radius based on the quality of the solutions found. When a better solution is discovered, the search radius is contracted, focusing the search on promising areas. Conversely, if a better solution is not found, the radius is expanded to explore a broader range of possibilities. In the constrained part of the algorithm, we apply sign restrictions using the knowledge of feasible space for $\Theta$. The pseudocode of the CANS algorithm is shown in Fig. 1C, where $NC$ is the number of perturbed solutions in each iteration, $J$ is the total number of iterations, ${\delta }_{u}$ is the noise upscaling coefficient, ${\delta }_{l}$ is the noise downscaling coefficient, and ${\sigma }_{max}$ is the maximum noise scale.

In each iteration $j\in \{1, 2, \dots , J\}$, $k$-th perturbed solution is generated by adding noise to the current best solution ${\Theta }^{\text{c}}=\left({\theta }_{0}^{c}, {\theta }_{1}^{c}, {\theta }_{2}^{c}, \dots , {\theta }_{6}^{c}\right)$:

8 $${{}^{k}\theta }_{i}^{\left(j\right)}={\theta }_{i}^{c}+{}{}^{k}{\epsilon }_{i} , \forall i=0, 1, \dots , 6\;\&\;k=\left\{1, 2, \dots , NC\right\}$$

where ${}^{k}{\epsilon }_{i}$ is the noise added to the $i$-th parameter for the $k$-th perturbed solution, sampled from a scaled standard normal distribution:

9 $${}^{k}{\epsilon }_{i} \stackrel{iid}{\sim } \mathcal{N}\left(0, {\sigma }^{2}\right) , \forall i=0, 1, \dots , 6\;\&\;k=\left\{1, 2, \dots , NC\right\}$$

where $\sigma$ is the noise scale. The sign function $\Phi$ is then applied to modify the parameters according to the existing domain restrictions:

10 $${{}^{k}\theta }_{i}^{{\prime}(j)}=\Phi \left({{}{}^{k}\theta }_{i}^{\left(j\right)}\right), \forall i=0, 1, \dots , 6\;\&\;k=\left\{1, 2, \dots , NC\right\}$$

11 $$\Phi \left({{}{}^{k}\theta }_{i}^{\left(j\right)}\right)=\left\{\begin{array}{c}{{}{}^{k}\theta }_{i}^{\left(j\right)},\;if\;i=\{0\} \\ \left|{{}{}^{k}\theta }_{i}^{\left(j\right)}\right|,\;if\;i=\{1, 2\} \\ -\left|{{}{}^{k}\theta }_{i}^{\left(j\right)}\right|,\;if\;i=\{3, 4, 5, 6\}\end{array}\right.$$

Sign function $\Phi \left(.\right)$ ensures that the candidate parameters generated in each iteration are feasible; that is, ${{}^{k}\theta }_{0}^{{\prime}(j)}\in {\mathbb{R}}$, ${{}^{k}\theta }_{1}^{{\prime}(j)},{{}{}^{k}\theta }_{2}^{{\prime}(j)}\in {\mathbb{R}}_{\ge 0}$, ${{}^{k}\theta }_{3}^{{\prime}(j)}, {{}{}^{k}\theta }_{4}^{{\prime}(j)},{{}{}^{k}\theta }_{5}^{{\prime}(j)},{{}{}^{k}\theta }_{6}^{{\prime}(j)}\in {\mathbb{R}}_{\le 0}$.

Next, all modified perturbed parameter vectors ${{}^{1}{\Theta }^{\prime}}^{\left(j\right)} ,{{{}{}^{2}\Theta }^{\prime}}^{\left(j\right)},\dots , {{}{}^{NC}{\Theta }^{\prime}}^{\left(j\right)}$ are evaluated, and the one that results in the smallest loss function value is selected (denoted as ${\Theta }_{min}^{\left(j\right)}$):

12 $${\Theta }_{\text{min}}^{\left(j\right)}=\underset{\Theta }{\text{argmin}}\left\{\mathcal{L}\left({{}{}^{1}{\Theta }^{\prime}}^{\left(j\right)}\right),\mathcal{L}\left({{{}{}^{2}\Theta }^{\prime}}^{\left(j\right)}\right),\dots ,\mathcal{L}\left({{}{}^{NC}{\Theta }^{\prime}}^{\left(j\right)}\right)\right\}$$

In each iteration, the objective function value evaluated at ${\Theta }_{\text{min}}^{\left(j\right)}$ is compared with the current best loss function value ${\mathcal{L}}^{c}$. If there is any improvement, the ${\mathcal{L}}^{c}$ value is updated, and the search radius is decreased by a downscaling factor of ${\delta }_{l}$; otherwise, the search space is expanded by an upscaling factor of ${\delta }_{u}$. To avoid overshooting, the scale parameter of the Gaussian distribution used to generate the noise is bound to be ${\sigma }_{max}$ at most (as detailed in Fig. 1C).

This optimization procedure is part of the OptiCS algorithm that is responsible for finding ${\Theta }^{*}$, which is the optimal parameter vector corresponding to the optimal loss function value ${\mathcal{L}}^{*}$.

Hyperparameter values of the CANS algorithm used in this study are listed in Supplementary Table S2.

#### Final predictive pipeline

Up until this point, we used the predictive pipeline $g$ to obtain the optimal parameter vector ${\Theta }^{*}$. To expedite the training process, we chose a simple pipeline $g$ based on logistic regression. The hyperparameters of this pipeline were largely fixed to enhance training speed. However, once the ${\Theta }^{*}$ is obtained, we transitioned to a more advanced pipeline for the final prediction, denoted as $G$. The primary distinctions between the two predictive pipelines, $g$ and $G$, are outlined below:

1. *Choice of classifier*: We used XGBoost in $G$ instead of logistic regression. XGBoost is a powerful machine learning algorithm for tabular data [29].
2. *Scaling*: We used robust scaling in $G$ to account for possible outliers. In this approach, the median is subtracted from the data, and the data is then scaled according to the interquartile range (IQR).
3. *Feature selection procedure*: In $g$, the reduced feature space size ($\mathcal{F}$) and tree maximum depth (${m}_{fs}$) were fixed to a predefined value. Here, in $G$, we allowed the model to find the optimal value through the tuning process. Therefore, we allowed for more flexibility in feature selection.
4. *Hyperparameter tuning approach*: $g$ utilized a grid search approach for the tuning procedure. However, due to the complexity of the main classifier and considering $\mathcal{F}$ and ${m}_{fs}$ as other hyperparameters to be tuned, using the same approach was not efficient anymore and would require us to try a vast combination of different values for the hyperparameters. Therefore, we incorporated Bayesian optimization to find the optimal hyperparameters for $G$.

We trained $G$ using the ${D}^{trainval}$ which is the training and validation sets combined. For the final assessment reported in the “Results” section, an untouched set ${D}^{test}$ was used.

### Hyperparameters

There are numerous hyperparameters used in this study. To summarize them and help achieve reproducibility of the results, we gathered and grouped them into two tables: (I) hyperparameters of the OptiCS algorithm (see Supplementary Table S2) and (II) hyperparameters of the final predictive pipeline $G$ (see Supplementary Table S3).

### Baseline models

Due to the lack of similar frameworks or algorithms in the literature, we could not benchmark the performance of the proposed algorithm against established standards. To address this, we developed three baseline models to evaluate the effectiveness of our algorithm.

The first two baseline models were designed to individually assess the contributions of dMRI and demographic data to the classification process. These models, named “dMRI Only” and “Demographics Only,” followed the same procedural steps as the proposed algorithm but each utilized only one of the datasets. This allowed us to determine the impact of feature exclusion (dMRI or Demographics) on predictive performance.

The third baseline model introduced a different scoring approach using principal component analysis (PCA). In this model, PCA was applied to standardized cognitive test scores, and the first principal component was extracted and mapped into the interval between 0 and 1 using a sigmoid function. The labeling process then followed the same approach as the proposed algorithm. This baseline can be seen as a simplified version of the proposed algorithm, where a linear combination of cognitive tests is used for scoring where the weights are obtained from the PCA loading vector corresponding to the first component. Here, the key distinction is that the parameter vector associated with the scoring function is not optimized.

In conclusion, the baseline models “dMRI Only” and “Demographics Only” provide benchmarks to evaluate the individual contributions of dMRI and demographic features, respectively. These models help us understand how each dataset performs in isolation and how their combination enhances the predictive capabilities of the proposed algorithm. On the other hand, the “PCA” baseline model assesses the effectiveness of optimization within the proposed algorithm.

### Evaluation

In this study, we divided the datasets into three independent subsets: training (3996), validation (893), and test set (908): The training set is applied to train the predictive pipeline $g$, the validation set is used to assess the performance of $g$, and the test set is an untouched partition of the data which is only used for the final assessment.

Different metrics were used to evaluate the algorithm. Terms were defined as follows:

True positives, or $tp$: Number of Positive-Agers that were predicted as Positive-Agers.

False positives, or $fp$: Number of Cognitive Decliners that were predicted as Positive-Agers.

True negatives, or $tn$ : Number of Cognitive Decliners that were predicted as Cognitive Decliners.

False negatives, or $fn$: Number of Positive-Agers that were predicted as Cognitive Decliners.

The evaluation metrics and their equations are

13 $$Accuracy=\frac{tp+tn }{tp+tn+fp+fn}$$

14 $$Precision=\frac{tp }{tp+fp}$$

15 $$Recall (sensitivity)=\frac{tp}{tp+fn}$$

16 $$Specificity=\frac{tn}{tn+fp}$$

17 $$F1-\text{score}=\frac{2\times precision\times recall}{Precision+recall}$$

## Results

In this section, we begin by analyzing the numerical results and comparing the performance of the proposed algorithm with that of the baseline models. Subsequently, we evaluate feature significance using SHapley Additive exPlanations (SHAP) values. SHAP values offer a comprehensive method for interpreting the contribution of individual features to the classification outcomes, thereby enabling us to identify which variables have the most significant impact on the algorithm’s predictions. Following this, we outline the validation steps undertaken to ensure the effectiveness of the scoring function. We then conduct a post-hoc analysis to explore the optimal scores generated by the proposed algorithm. This analysis includes a detailed comparison between Positive-Agers and Cognitive Decliners with respect to dMRI features and demographic data. Finally, we address the question: “Is it possible to predict future trajectories using only the baseline visit data?”.

### Numeric results for cognitive classification

Here, we present a comparative analysis of the proposed algorithm against baseline models, with the results summarized for different quantile thresholds in Table 1. We categorized our findings into the following subsections:

**Table 1:** Classification results for predicting cognitive classes

Threshold	Model	AUC	Accuracy	Precision	Recall	Specificity	F1	*N*^‡^
${\varvec{q}}$= 0.10	Algorithm	0.80*	**0.72**	**0.70**	0.75	**0.68**	0.72	1175
	dMRI Only	0.69	0.65	0.65	**0.86**	0.35	**0.74**	1382
	Demo Only^†^	0.76	0.69	0.66	0.74	0.65	0.70	1162
	PCA	0.76	0.67	0.66	0.68	0.67	0.67	1163
${\varvec{q}}$= 0.15	Algorithm	**0.83**	**0.72**	**0.74**	0.71	**0.74**	0.72	1743
	dMRI Only	0.65	0.59	0.57	0.62	0.55	0.60	1750
	Demo Only	0.76	**0.72**	0.72	**0.75**	0.68	**0.73**	1732
	PCA	0.78	0.71	0.71	0.72	0.70	0.72	1762
${\varvec{q}}$= 0.20	Algorithm	**0.81**	**0.72**	0.72	**0.74**	0.70	**0.73**	2296
	dMRI Only	0.68	0.62	0.64	0.60	0.64	0.62	2289
	Demo Only	0.75	0.67	0.67	0.71	0.64	0.70	2328
	PCA	0.79	0.71	**0.73**	0.70	**0.72**	0.72	2348
${\varvec{q}}$= 0.25	Algorithm	**0.80**	**0.74**	**0.72**	**0.79**	**0.69**	**0.75**	2864
	dMRI Only	0.62	0.59	0.58	0.63	0.55	0.60	2901
	Demo Only	0.75	0.68	0.67	0.74	0.62	0.70	2877
	PCA	0.77	0.72	0.71	0.76	0.68	0.73	2901

^*^Values in bold font are the best result in each metric for a given quantile threshold

^‡^Final sample size (training + validation + test) after labeling and exclusion of the Normal-Aging group

^†^Demo = Demographics

#### Optimal quantile threshold

**Fig. 2:**
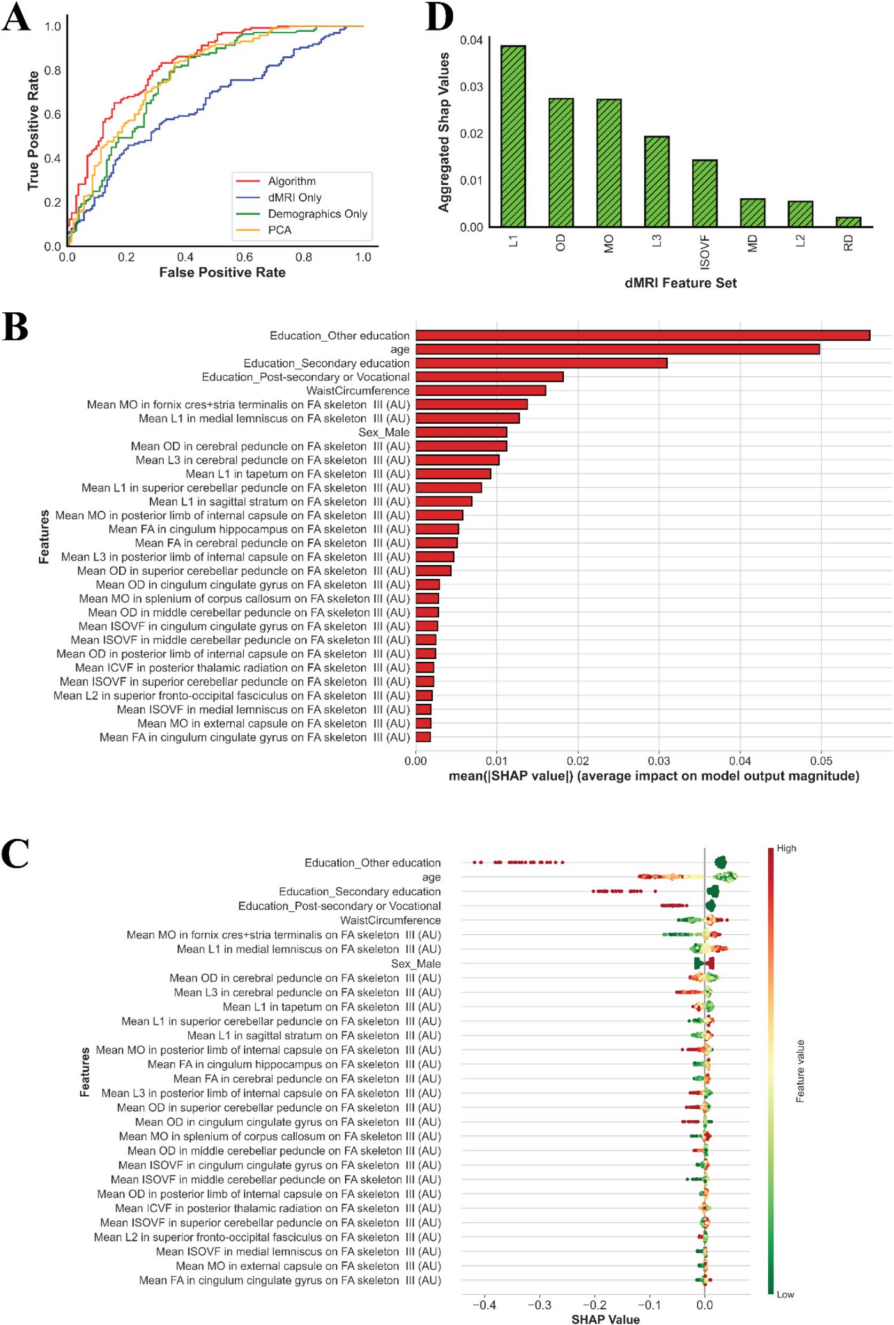
Evaluating the performance of the algorithm and feature contribution. **A** ROC curves for the algorithm and baseline models for $q$ = 0.15. **B** Feature importance for the top 30 features using the mean of absolute SHAP values. **C** SHAP values for the top 30 features. **D** Total aggregated importance of dMRI feature sets

Using a quantile threshold of $q$ = 0.15 yielded the highest AUC value, reaching 83% (see Fig. 2A for the ROC curve comparison). This suggests that increasing the threshold further introduces noise into the labeling procedure by including individuals who may not belong to the “Cognitive Decliner” or “Positive-Ager” class and may be Normal-Agers. Thus, $q$ = 0.15 appears to be the optimal balance, minimizing the inclusion of noise and enhancing the classification accuracy. In this setting, we achieved an accuracy of 72%, a precision of 74%, a recall value of 71%, a specificity of 74%, and an F1-score of 72%.

#### Performance consistency

The proposed algorithm consistently outperformed the baseline models in terms of AUC, regardless of the chosen quantile threshold. Additionally, the proposed algorithm demonstrated superior performance across other metrics within each quantile category. It is important to note that the baseline models share foundational similarities with the proposed algorithm, which means they may not serve as entirely independent benchmarks. Nevertheless, in the absence of relevant frameworks in the literature, these designed baselines are valuable for assessing the methodology’s effectiveness, particularly in terms of feature inclusion and the optimization loop.

#### Feature inclusion

When comparing the full algorithm with the “dMRI Only” and “Demographics Only” baseline models, it is evident that the combination of dMRI and demographic data is essential for achieving maximum performance. While utilizing only dMRI features and ignoring demographic characteristics significantly decreased the performance metrics across all threshold categories, relying solely on demographic features did not result in as severe an accuracy loss as the “dMRI Only” model. However, after adding the dMRI features to the “Demographics Only” model, the AUC improved by 7% and specificity also increased by 6%. Please refer to the Supplementary Text: Sect. 3A for explanations about why the exclusion of the dMRI subset was conceptually and methodologically not tenable.

#### Optimization

Comparing the results between the “PCA” model and the full algorithm shows that using the optimal parameter vector enhanced overall performance.

### Contribution of features in cognitive classification

We used SHAP values to quantify the contribution of features for the final prediction. Figure 2B illustrates the average of absolute SHAP values for the top 30 features. The higher the mean absolute SHAP value, the larger the magnitude of the average impact will be for a particular feature. Among the demographic variables, education, age, waist circumference, and sex were the most influential features. In the dMRI dataset, the top six features were “mean MO in fornix cres + stria terminalis,” “mean L1 in medial lemniscus,” “mean OD in cerebral peduncle,” “mean L3 in cerebral peduncle,” “mean L1 in tapetum,” and “mean L1 in superior cerebellar peduncle.”

Plotting SHAP values can also be insightful because it helps us understand the direction of the effect for each variable, as well as the spread of the effect. Figure 2C shows the SHAP values for the top 30 features. There were several interesting findings: (I) Individuals with a low level of education were less likely to be “Positive-Agers.” This negative effect became stronger as the level of education decreased. (II) Aligned with our expectations, general cognitive performance decreased as people age. This can be concluded since older individuals have negative SHAP values showing a decrease in the predicted probability of being a Positive-Ager. (III) Men were more likely to be Positive-Agers. (IV) As waist circumference increases, the predicted probability of being in the “Positive-Ager” class increases in general (see the “Discussion” section for a more comprehensive analysis). (V) Individuals with higher values of “mean MO in fornix cres + stria terminalis” and “mean L1 in medial lemniscus” are more likely to be Positive-Agers, while individuals with higher values of “mean OD in cerebral peduncle” are less likely to be Positive-Agers (see detailed analysis in the “Analysis of dMRI attributes” section).

We plotted the aggregated feature importance, which helped us identify the effect of various dMRI attributes. For this purpose, we summed up the mean absolute SHAP values corresponding to any of the dMRI attributes (see Fig. 2D). We also plotted the aggregated SHAP values for various regions of interest in the brain, which is shown in Supplementary Fig. S2.

### Cognitive scoring function

Despite the intricacies of the internal workings of the algorithm, the final output is a straightforward and interpretable mathematical equation. This equation can be utilized to quantify an individual’s cognitive score based on longitudinal cognitive assessments. This is expressed as

18 $$\gamma =f\left(X \right| {\Theta }^{*})=\sigma \left({\Theta }^{*}X\right)=\frac{1}{1+{e}^{-{\Theta }^{*}X }}$$

where $${\Theta }^{*}X$$ is

19 $${\Theta }^{*}X=-0.622+1.764\left(\frac{F{I}_{1}-6.86}{1.98}\right)+ 2.198\left(\frac{F{I}_{3}-6.79}{2.03}\right)-0.349\left(\frac{PM{M}_{1}-1.40}{0.62}\right)-1.371\left(\frac{PM{M}_{3}-1.41}{0.61}\right)-0.633\left(\frac{R{T}_{1}-6.32}{0.18}\right)-1.191\left(\frac{R{T}_{3}-6.40}{0.17}\right)$$

Here, $F{I}_{1}, F{I}_{3}, \dots , R{T}_{3}$ are the original exam values, which are standardized in parentheses with the corresponding means and standard deviations found during the training process. The labels are then assigned based on the lower and upper bounds obtained by the algorithm:

20 $$y=\left\{\begin{array}{c}`\!\ \!`Cognitive\;Decliner\!",if\gamma\leq0.004\\`\!\ \!`Positive-Ager\!",if\gamma\geq0.986\\`\!\ \!`Normal-Ager\!", otherwise\end{array}\right.$$

**Fig. 3:**
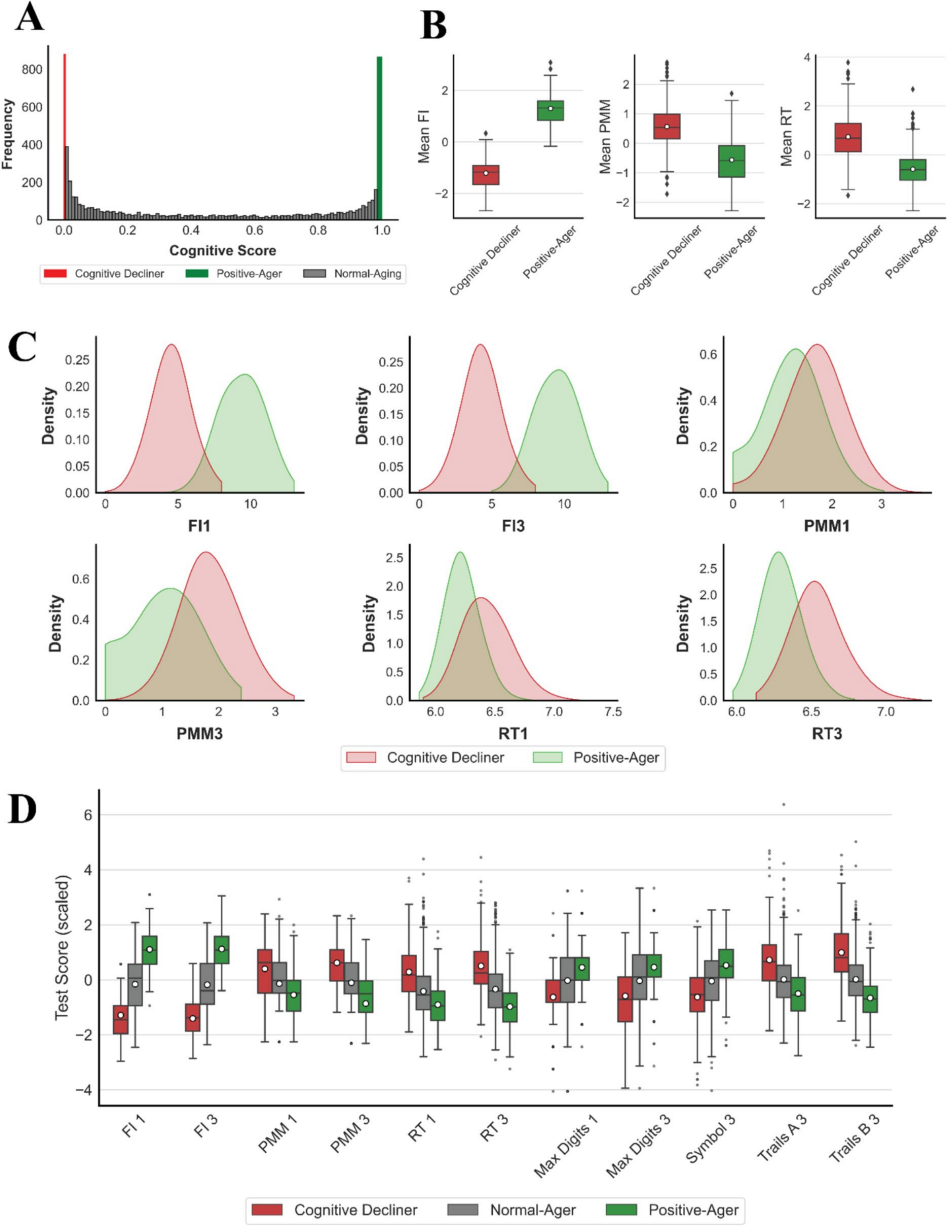
Comparison of cognitive exams between different cognitive classes. **A** Histogram of optimal cognitive scores ($\gamma$). Positive-Agers and Cognitive Decliners are at the two ends of the score range. **B** Boxplot of the average cognitive exams of ${t}_{1}$ and ${t}_{3}$ for Positive-Agers and Cognitive Decliners. **C** Estimated kernel density plots of cognitive tests for Positive-Agers and Cognitive Decliners at each visit, separately. **D** Boxplot of cognitive tests for an independent sample of 1482 participants who were not included in any part of the study (FI, fluid intelligence; PMM, pairs matching memory; RT, reaction time)

There are several interesting facts about the yielded cognitive scoring function. First, by comparing the coefficient magnitudes at ${t}_{1}$ and ${t}_{3}$ for each cognition test, it can be observed that the algorithm emphasized more on the test results collected on the third visit (${t}_{3}$). This makes sense because the dMRI attributes are also collected at the third visit. In addition, time point ${t}_{3}$ provides the most recent assessment of participants’ cognitive performance. The algorithm already made proper adjustments accordingly. Second, the lower and upper bounds proposed by the algorithm are very close to the two ends of the cognitive scores’ interval (i.e., [0, 1]). This highlights that the algorithm effectively determines the scores, ensuring that the extreme groups are positioned at the very ends of the interval. It assigns labels only when there is clear evidence for an individual to belong to a specific cognitive group, indicating algorithm robustness. Figure 3A shows the histogram of cognitive scores calculated for the participants in this study. It can be observed that Positive-Agers and Cognitive Decliners are at the two ends of the score’s interval, depicting a perfect separation. Third, according to weight magnitudes, fluid intelligence is the most influential cognitive test, as the weight associated with this exam is the largest in absolute value. The RT and PMM tests were the second and third most influential tests. Fourth, the baseline score for someone performing the same as the average population on all tests is $\sigma$($-0.622)\approx 0.35$. This value seems reasonable, as we generally anticipate a decline in cognitive performance over time in the population. Therefore, having the baseline score closer to the “Cognitive Decliner” group makes sense.

The proposed cognitive scoring system offers several benefits. First, although we excluded the middle group (i.e., “Normal-Agers”) and focused on distinguishing between two extreme cognitive classes, the framework provides a continuous scoring mechanism that captures the entire range of cognitive performance. Specifically, if the computed cognitive score falls between 0.004 and 0.986, the individual belongs to the “Normal-Aging” group. Second, the current configuration maps cognition test results to a restricted interval between 0 and 1. This makes the score an interpretable metric that is easy to compare across individuals in a population. Third, the score’s range is fixed and independent of the population or cognitive exam values observed. This provides researchers with a mechanism to make comparisons across different populations concerning the distribution of cognitive performances and their characteristics. This, however, requires validation on independent samples first.

### Sensitivity analyses: validation of scoring system

Here, we examined whether the proposed cognitive scoring system produces reasonable scores or not. For this purpose, we conducted two sets of analyses. First, we compared the cognitive tests for individuals labeled as “Positive-Ager” against those identified as “Cognitive Decliners” by the algorithm. This assessment checks whether there is a clear separation between two cognitive classes in terms of cognitive performance or not, regardless of any additional input features. Table 2 summarizes the cognitive test’s mean and standard deviation at time points ${t}_{1}$ and ${t}_{3}$ for the two cognitive groups. An independent *t*-test was conducted to examine the difference in the mean levels. The *P*-values for all tests were significant, and the effect size calculated depicted a large effect size. This clearly indicates that the two classes significantly differ with respect to cognitive performance and verifies the algorithm’s effectiveness in creating such a separation. One interesting observation is that, in moving from ${t}_{1}$ to ${t}_{3}$, the absolute difference in the mean levels of the two classes and the corresponding effect size increases. This implies that the cognitive gap between the two classes increases as the population ages. The distribution of mean cognitive exams and individual tests between the two cognitive classes are shown in Fig. 3B, C, respectively. We noticed that FI tests were most effective in creating such a separation. This is aligned with our previous finding in the “Cognitive scoring function” section, where FI tests had the largest weight magnitudes in the optimized parameter vector found by the algorithm.

**Table 2:** Descriptive statistics of cognitive tests for “Positive-Agers” vs. “Cognitive Decliners”

Test	Cognitive Decliner	Positive-Ager	Absolute mean difference	*P*-value (effect size)
FI1	4.54 ± 1.31^‡^	**9.41**^†^ ± 1.48	4.87	< 1e-5 (− 3.5)*
FI3	4.29 ± 1.31	**9.46** ± 1.42	5.17	< 1e-5 (− 3.8)
PMM1	**1.65** ± 0.58	1.15 ± 0.60	0.50	< 1e-5 (0.9)
PMM3	**1.85** ± 0.49	0.97 ± 0.62	0.88	< 1e-5 (1.6)
RT1	**6.43** ± 0.20	6.22 ± 0.14	0.21	< 1e-5 (1.2)
RT3	**6.54** ± 0.17	6.30 ± 0.13	0.24	< 1e-5 (1.7)
Mean FI	4.41 ± 1.03	**9.43** ± 1.14	5.02	< 1e-5 (− 3.5)
Mean PMM	**1.75** ± 0.42	1.06 ± 0.43	0.69	< 1e-5 (0.9)
Mean RT	**6.49** ± 0.15	6.26 ± 0.11	0.23	< 1e-5 (1.2)

^*^P-value for the independent *t*-test. Values in parentheses are the Hedge’s *g* effects

^‡^Mean ± std. deviation

^†^The bold values show the highest mean value for each test

For the second set of validation experiments, we utilized an independent sample comprising cognitive test results from 1482 participants who were not included in any part of the study due to the unavailability of dMRI data. To enhance the robustness of the analysis, we incorporated additional cognitive exams such as the symbol digit substitution test, the maximum digits remembered test (i.e., numeric memory test), and the trail-making tests A and B (see Supplementary Text: Sect. 3B for further details on the definition of new exams). Using the same scoring function, we assigned cognitive labels to each participant. Figure 3D displays the boxplots for each cognitive test corresponding to this independent sample. As before, a clear distinction was observed between the mean levels for each cognitive exam.

In conclusion, both sets of analysis corroborate the algorithm’s effectiveness in distinguishing between the cognitive classes of “Cognitive Decliner” and “Positive-Ager.”

### Analysis of demographic characteristics

The summary statistics of demographic attributes for the two cognitive classes can provide valuable insights and is summarized in Table 3. We plotted the distribution of the most important demographic features, including education, age, waist circumference, and sex in Supplementary Fig. S3.

**Table 3:** Descriptive statistics for demographic features

Variable	Range	Cognitive Decliner (879)	Positive-Ager (864)	*P*-value (effect size)
Age at baseline	[55, 70]	61.69 ± 3.96*	59.88 ± 3.46	**0.0001**^‡^ (+ 0.49)^§^
BMI	[10, 31]	18.03 ± 2.78	18.44 ± 2.88	**0.0021** (− 0.15)
Waist circumference	[60, 138]	88.28 ± 11.66	90.14 ± 11.69	**0.0009** (− 0.16)
Education (other education)^†^	{0, 1}	15.8% (139)^⁜^	0.2% (2)	**0.0001**
Education (post-secondary or vocational)	{0, 1}	18.4% (162)	11.2% (97)	**0.0001**
Education (secondary education)	{0, 1}	15.4% (135)	4.4% (38)	**0.0001**
Handedness (left-handed)	{0, 1}	10.5% (92)	8.3% (72)	0.1490
Handedness (right-handed)	{0, 1}	87.6% (770)	90.7% (784)	**0.0422**
Household income (middle class)	{0, 1}	27.3% (240)	31.5% (272)	0.0626
Household income (under class)	{0, 1}	22.8% (200)	8.7% (75)	**0.0001**
Household income (upper class)	{0, 1}	3.0% (26)	7.9% (68)	**0.0001**
Household income (upper-middle class)	{0, 1}	14.0% (123)	30.6% (264)	**0.0001**
Sex (male)	{0, 1}	46.4% (408)	60.4% (522)	**0.0001**
Skin color (olive-skinned)	{0, 1}	21.4% (188)	18.1% (156)	0.0915
Skin color (pale-skinned)	{0, 1}	78.2% (687)	81.8% (707)	0.0635
Tobacco type (hand-rolled cigs)	{0, 1}	0.5% (4)	0.3% (3)	1.0000
Tobacco type (manufactured cigs)	{0, 1}	2.3% (20)	1.5% (13)	0.3151
Tobacco type (non-smoker)	{0, 1}	96.9% (852)	97.6% (843)	0.5020
Tobacco use (prior smoker)	{0, 1}	36.4% (320)	35.0% (302)	0.5603
Tobacco use (smoker)	{0, 1}	3.9% (34)	4.2% (36)	0.8450

^*^For continuous variables: mean ± std. deviation

^‡^Bold values show significance (*P*-value < 0.05)

^§^Hedge’s *g* effect size in parentheses

^†^Baseline level of categorical variables is not shown in this table but could be extracted from total participants or proportion in cognitive class

^⁜^For binary variables: percentage (number of participants)

For a detailed analysis of sex dimorphism in demographic features, please refer to Supplementary Table S4.

### Analysis of dMRI attributes

**Fig. 4:**
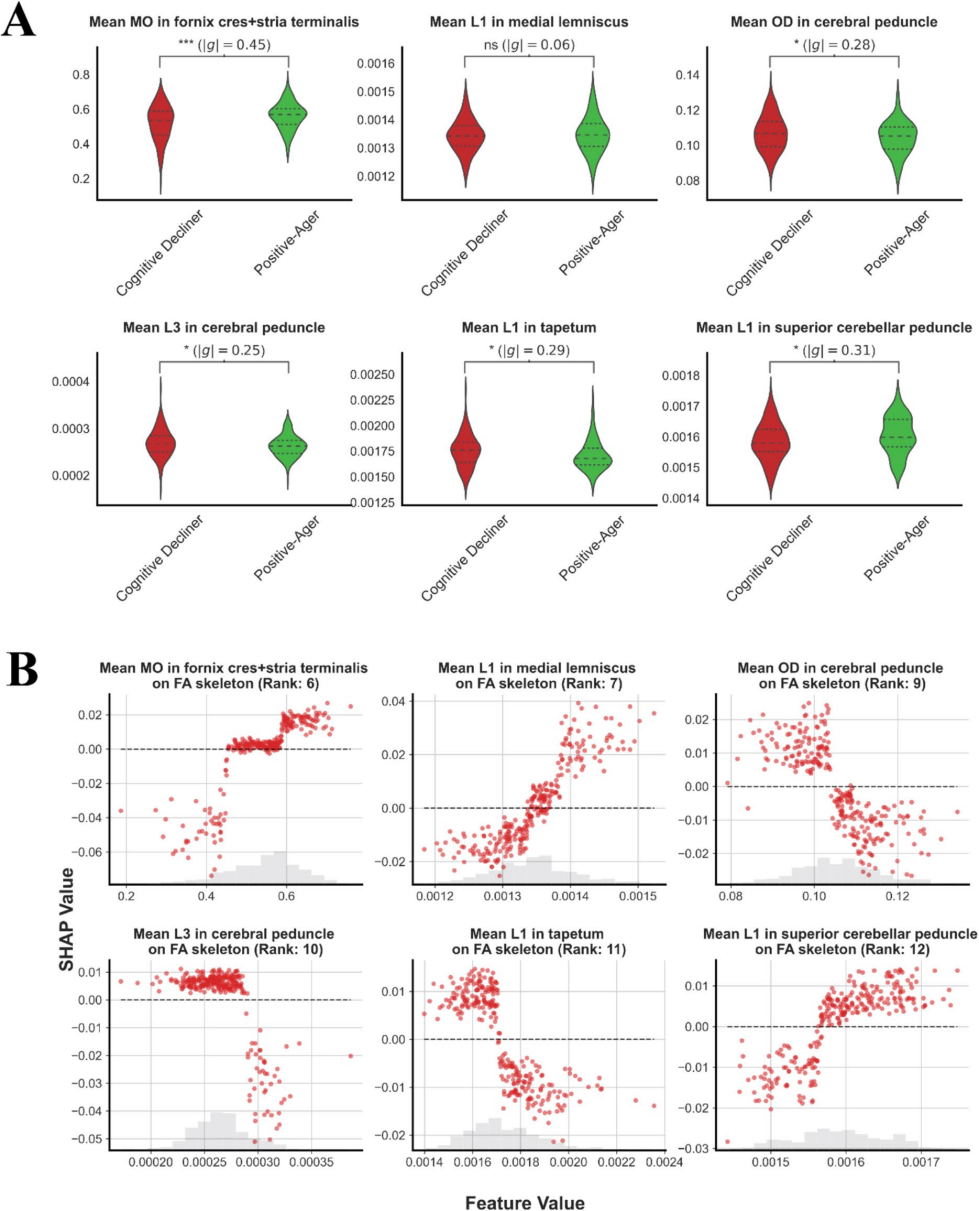
Analysis of the top six dMRI features using participants in the test set. **A** Violin plot of top six dMRI attributes for Positive-Agers and Cognitive Decliners. An independent *t*-test was conducted, and the *P*-values are reported on top of each violin plot (***P < 0.001; **P < 0.01; *P < 0.05; ns, non-significant). Values in parentheses are the absolute values of Hedge’s *g* effect. **B** Scatter plot of top six dMRI attributes against their SHAP values. The rank of a feature represents its relative position among all existing variables based on its importance

dMRI is one of the primary feature sets employed by the classifier to differentiate between the “Cognitive Decliner” and “Positive-Ager” classes. To elucidate the differences in dMRI attributes across these groups, we compared the values of the top dMRI features. Figure 4A presents violin plots for the top six regions of DTI data, highlighting the disparities between the two groups. An independent *t*-test conducted on participants in the test set ($n$ = 270) revealed significant differences at the 5% significance level between the mean levels of these cognitive classes for each dMRI attribute, except for “mean L1 in medial lemniscus,” where the mean values were nearly identical (difference of 3.6e-6); hence, the test was not able to detect mean differences. Boxplots for the remaining regions are provided in Supplementary Fig. S4.

Having examined the differences between the two optimal classes identified by the algorithm, it is insightful to explore the relationship between dMRI attributes and the likelihood of positive-aging as the next step. Figure 4B presents scatter plots of SHAP values against the values of the top dMRI features. The analysis uncovered that mean MO in the fornix + stria terminalis, mean L1 in the medial lemniscus, and mean L1 in the superior cerebellar peduncle exhibit a positive correlation with the likelihood of positive aging. Conversely, the other three attributes, including mean OD and mean L3 in the cerebral peduncle and mean L1 in the tapetum, show a negative correlation. Below, we explore the details of the direction and magnitude of the impact for each of these dMRI attributes:

#### Mean MO in fornix + stria terminalis on FA skeleton

We observed that mean MO values in the fornix and stria terminalis tracts greater than 0.58 were associated with approximately a 2% increased likelihood of being a Positive-Ager. Conversely, values smaller than 0.45 decreased this likelihood by up to 7% in some participants.

#### Mean L1 in medial lemniscus on FA skeleton

There is a positive linear relationship for values larger than 0.00135, with an up to 4% increase in the likelihood of positive aging. Values smaller than this threshold are associated with a decrease in the likelihood of positive aging, with a mean impact of around 1%.

#### Mean OD in cerebral peduncle on FA skeleton

Values larger than 0.104 were associated with a decrease in the positive aging likelihood, while values smaller than this threshold increased this likelihood. This impact can be up to 2.5% in both directions.

#### Mean L3 in cerebral peduncle on FA skeleton

Values less than 0.0029 in the crus cerebri did not have a significant impact (maximum impact of 1%). However, values higher than this threshold were associated with a decrease in the likelihood of positive aging by up to 5%.

#### Mean L1 in tapetum on FA skeleton

Axial diffusivity values less than 0.0017 were associated with an increased positive aging likelihood, while values higher than this threshold had a negative impact of up to 2%.

#### Mean L1 in superior cerebellar peduncle on FA skeleton

Values higher than 0.00157 are positively correlated with the likelihood of positive aging, whereas values less than this cutoff were associated with a decrease in this likelihood by up to 3%.

The scatter plot of the impact for the remaining dMRI attributes is shown in Supplementary Fig. S5.

### Longitudinal analysis of cognitive trajectories

Although the proposed methodology has provided a powerful yet straightforward mechanism to quantify individuals’ cognitive performance, equipping the algorithm with tools to study cognitive trajectories and rates of change may offer additional insights into how cognitive capabilities evolve over time.

Considering the scoring system proposed, we can decompose it into three independent components:

21 $${\Theta }^{*}X={\theta }_{0}^{*}+{S}_{1}^{adj}+{S}_{3}^{adj}$$

Here, ${S}_{1}^{adj}$ and ${S}_{3}^{adj}$ are called adjusted scores at ${t}_{1}$ and ${t}_{3}$, respectively. The term “adjusted” refers to the fact that scores are computed based on the optimal weights obtained from the algorithm’s output:

22 $${S}_{1}^{adj}={\theta }_{1}^{*} F{I}_{1}^{s}+{\theta }_{3}^{*} PM{M}_{1}^{s}+{\theta }_{5}^{*}R{T}_{1}^{s}$$

23 $${S}_{3}^{adj}={\theta }_{2}^{*} F{I}_{3}^{s}+{\theta }_{4}^{*} PM{M}_{3}^{s}+{\theta }_{6}^{*}R{T}_{3}^{s}$$

**Fig. 5:**
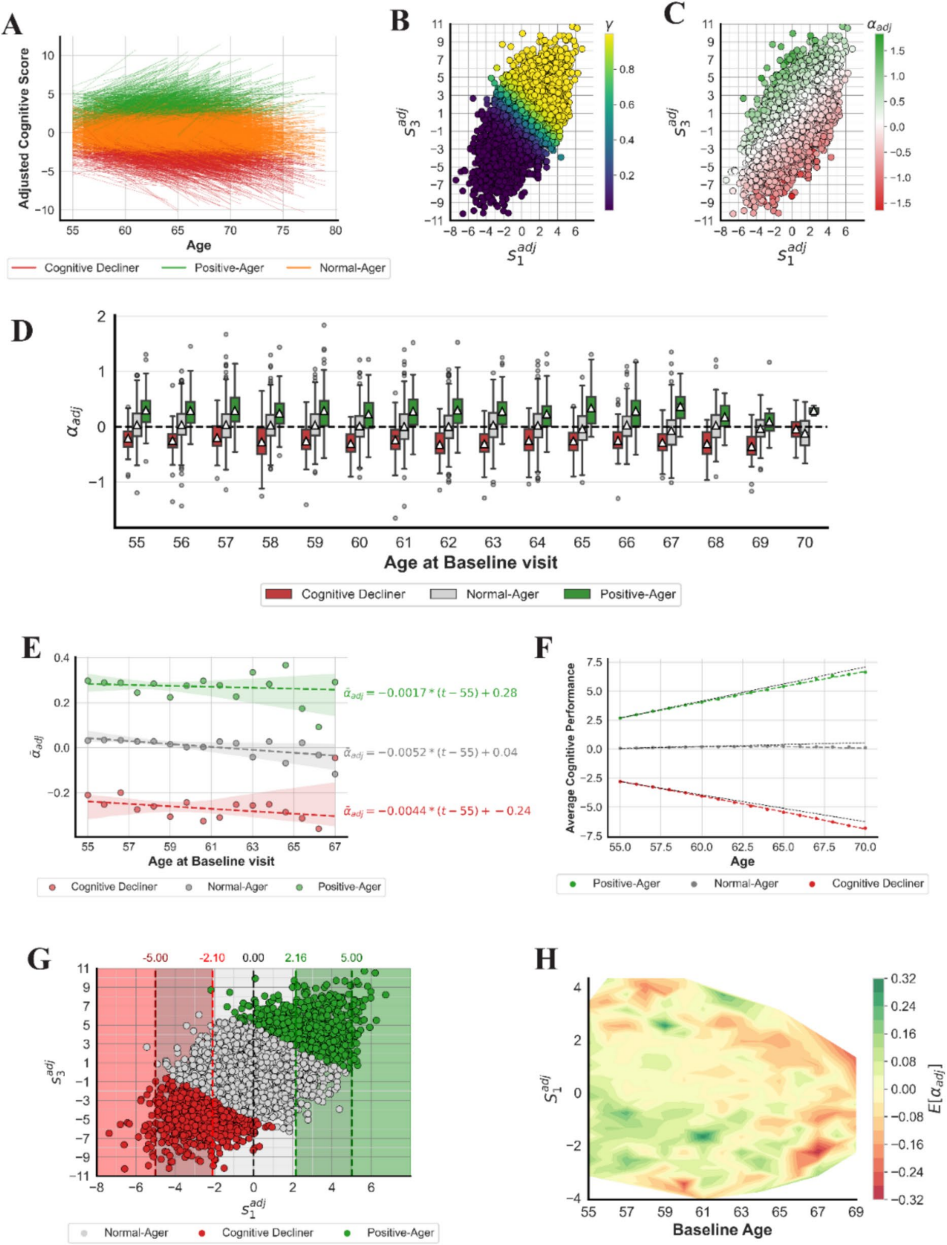
Analysis of cognitive trajectory and the corresponding slopes. **A** Cognitive trajectories plotted based on ${S}_{1}^{adj}$ and ${S}_{3}^{adj}$. The color of each line represents the corresponding cognitive class to which an individual is assigned (red: Cognitive Decliner; orange: Normal-Ager, and green: Positive-Ager). **B** Scatterplot of ${S}_{1}^{adj}$ and ${S}_{3}^{adj}$ with respect to the cognitive score, $\gamma$, calculated. **C** Scatter plot of ${S}_{1}^{adj}$ and ${S}_{3}^{adj}$ with respect to adjusted trajectory slopes, ${\alpha }_{adj}$. **D** Boxplot of adjusted trajectory slopes at different levels of baseline age. **E** Scatter plot and regressed line of the average adjusted trajectory slope against baseline age. **F** Constructed average cognitive trajectory curve for different cognitive groups. Dashed lines are straight lines that aim to better visualize the main trajectory path. **G** Scatter plot of ${S}_{1}^{adj}$ and ${S}_{3}^{adj}$ with respect to the cognitive classes. **H** The heatmap of the expected slope, given the initial adjusted score and age (${S}_{1}^{adj}$, adjusted score at ${t}_{1}$; ${S}_{3}^{adj}$, adjusted score at ${t}_{3}$; ${\alpha }_{adj}$, adjusted trajectory slope)

We plotted the cognitive trajectories using ${\text{S}}_{1}^{adj}$ and ${\text{S}}_{3}^{adj}$ in Fig. 5A. The figure illustrates a clear separation between different cognitive groups in terms of both the trajectory’s starting points and its slope: Positive-Agers are located in the positive zone and generally exhibit more positive slopes, indicating an upward trend in their cognitive trajectories as expected. Conversely, Cognitive Decliners are situated in the negative zone and demonstrate a downward trend. Individuals classified as Normal-Agers fall in the middle, with slopes close to zero, scattered around the zero mark.

After this reformulation, we can rewrite the definition of Cognitive Decliners as below:

24 $$\frac{1}{1+{e}^{-\left({\theta }_{0}^{*}+{S}_{1}^{adj}+{S}_{3}^{adj}\right)}}\le lb$$

which can be simplified as

25 $${S}_{3}^{adj}\le -{\theta }_{0}^{*}-{S}_{1}^{adj}+\text{ln}\left(\frac{lb}{1-lb}\right)$$

After substituting ${\theta }_{0}^{*}$ and $lb$ with the optimal values shown in (19) and (20), that is ${\theta }_{0}^{*}=-0.622, lb=0.004$, we have the optimal inequality corresponding to the definition of Cognitive Decliners:

26 $${S}_{3}^{adj}\le -4.9-{S}_{1}^{adj}\;OR\;{S}_{3}^{adj}+{S}_{1}^{adj}\le -4.9$$

This implies that if the sum of adjusted scores from the two visits is − 4.9 or lower, the participant is classified as a Cognitive Decliner. Equivalently, for a given initial visit with an adjusted score ${S}_{1}^{adj}=s$, the highest possible score in the next visit that will still categorize the individual as a Cognitive Decliner is $-4.9-s$.

Applying the same procedure to the “positive-aging” definition, we have

27 $${S}_{3}^{adj}\ge -{\theta }_{0}^{*}-{S}_{1}^{adj}+\text{ln}\left(\frac{ub}{1-ub}\right)$$

Substituting $ub=0.986$ and ${\theta }_{0}^{*}=-0.622$ will result in the following inequality:

28 $${S}_{3}^{adj}\ge 4.9-{S}_{1}^{adj}\;OR\;{S}_{3}^{adj}+{S}_{1}^{adj}\ge 4.9$$

This means that if the sum of adjusted scores from the two visits is 4.9 or higher, the participant is classified as a Positive-Ager. Equivalently, for a given initial visit with an adjusted score ${S}_{1}^{adj}=s$, the lowest possible score in the next visit that will place the individual into the Positive-Ager class is $4.9-s$. Supplementary Fig. S6 shows the corresponding line equations.

Another advantage of the reformulation in Eq. (21) is that we are now able to analyze the rate of change in cognitive performance for a given participant using Eq. (29).

29 $${\alpha }_{adj}=\frac{{\text{S}}_{3}^{adj}-{\text{S}}_{1}^{adj}}{\Delta t}$$

This new variable, ${\alpha }_{adj}$, is termed the adjusted trajectory slope. To understand the rationale behind using the adjusted trajectory slope instead of other forms, refer to Supplementary Fig. S7. This figure compares various approaches to computing the rate of change and demonstrates that optimal weights in the adjusted scores provide the best separation between cognitive classes. The relation between ${S}_{1}^{adj}$ and ${S}_{3}^{adj}$ with cognitive score ($\gamma$) and trajectory slope (${\alpha }_{adj}$) is shown in Fig. 5B, C, respectively. For a deeper analysis of the relationship between the computed cognitive scores and adjusted trajectory slope, refer to Supplementary Fig. S8.

Studying the trajectory slope in different age groups may provide additional insights. For this purpose, we plotted the boxplot of the adjusted trajectory slope for cognitive groups at different levels of baseline age in Fig. 5D. As the plot suggests, there is a significant difference in the mean level of adjusted cognitive slopes across the three cognitive groups. Additionally, we observed a consistent pattern: on average, regardless of baseline age, individuals in the positive-aging group have a positive slope, individuals in the Cognitive Decliner group have a negative slope, and those in the Normal-Aging group have a slope value close to zero. This observation leads to the question, “How does the slope change as people age, then?”. Figure 5E addresses this by plotting the average adjusted trajectory slope (${\overline{\alpha }}_{adj}$) against baseline age. We used robust linear regression to capture the linear trend in the data while ignoring outlier data points. Several interesting observations emerged: First, on average, trajectory slopes decreased as people age. Also, this figure suggests that individuals with improving trajectories experience less improvement over time, while those with declining cognitive performance experience greater cognitive decline. Second, comparing the age coefficient in the regressed lines, we noticed that the magnitude of changes in Cognitive Decliners was about 2.5 times that of Positive-Agers, indicating that the change in the rate of cognitive decline was significantly steeper than that of cognitive improvement as people age.

### Constructing the average cognitive trajectory curve

In Fig. 5E, we analyzed the average trajectory slope given a baseline age. The points on that plot can be viewed as the derivative of a primary average cognitive trajectory curve at different age levels (i.e., years of age). Our aim here is to construct the cognitive trajectory curve using the observed derivative values for each cognitive class. Suppose Eq. (30) represents the line equation for the trajectory slope:

30 $${\overline{\alpha }}_{adj}=mt+b$$

By integrating this equation, we can obtain the average cognitive trajectory curve, denoted as $\mathcal{C}$:

31 $$\mathcal{C}=\int (mt+b)dt=\frac{m{t}^{2}}{2}+bt+u$$

where $u$ is a constant and was replaced with the average ${S}_{1}^{adj}$ at the initial baseline age for this subset of UK Biobank data (55 years). Figure 5F shows the estimated average cognitive trajectory for each group based on the observed data. The dashed lines are plotted alongside the curves to compare them with the corresponding straight lines, providing a clearer visualization of the changes in cognitive performance over time. This figure suggests that as people age, the gap in cognitive capability between Positive-Agers and Cognitive Decliners increases. This observation aligns with our previous findings in the “Sensitivity analyses: validation of scoring system” section, where we compared the absolute difference in mean cognitive exam levels at ${t}_{1}$ versus ${t}_{3}$ (see Table 2). The resulting mathematical equations for each cognitive class are presented in Supplementary Text: Sect. 3C.

### Predict cognitive trajectory on the first visit

This study utilizes longitudinal data. However, deriving insights solely from the baseline visit to forecast future trajectories could be beneficial in identifying individuals’ cognitive categories in advance. To this end, we initially plotted ${\text{S}}_{1}^{adj}$ and ${\text{S}}_{3}^{adj}$ in relation to different cognitive classes, as illustrated in Fig. 5G. Several significant observations emerged. First, individuals with a positive initial adjusted score were highly unlikely to be classified as “Cognitive Decliners,” while those with a negative ${\text{S}}_{1}^{adj}$ are rarely “Positive-Agers.” Second, all individuals with ${\text{S}}_{1}^{adj}\ge 5$ were identified as “Positive-Agers” regardless of their subsequent assessments, and those with ${\text{S}}_{1}^{adj}\le -5$ were predominantly “Cognitive Decliners,” with only one exception. This pattern holds true for the interval between visits in this study. The subsequent boundaries, ${S}_{1}^{adj}=2.16$ and ${S}_{1}^{adj}=-2.10$, shown in Fig. 5G, were determined by minimizing the Gini index and representing the optimal split points for identifying cognitive groups. Individuals with initial adjusted scores higher than 2.16 are more likely to be “Positive-Agers,” while those with scores lower than − 2.10 are more likely to be “Cognitive Decliners.” Participants with scores between these thresholds are mostly “Normal-Agers.”

Next, we investigated the possibility of estimating the trajectory slope using the baseline age and initial adjusted score, ${S}_{1}^{adj}$ (see Fig. 5H). The plot presents a heatmap of the expected slope for a given initial adjusted score ($s$) and baseline age ($t$). The expected slope at each point is computed based on the mean slope observed for participants aged $t$, whose ${S}_{1}^{adj}$ was within 0.25 of the value $s$ (i.e., within the interval $[s-0.25, s+0.25]$). Furthermore, we imposed a requirement of having at least ten participants in that range to estimate the mean slope. Several notable observations can be drawn from this figure. First, individuals with negative scores in their 5th decade of life (i.e., 50s) are more likely to show improvement over time compared to those with similar scores in their late 60s. Second, a general decline is observed in the late 60s, irrespective of the initial score, when compared to individuals in their 50s. Third, participants with very high scores (${S}_{1}^{adj}$ values close to 4) tend to experience a general decline in their cognitive scores over time. This may suggest that improvement beyond such high scores is improbable within this age range, suggesting that individuals either maintain the same level of cognitive performance or experience a decline over time.

As the final step, we utilized all available baseline data, including demographic information and exam results, to predict the cognitive classes: “Positive-Ager,” “Cognitive Decliner,” and “Normal-Ager.” A decision tree model was trained on the same data used in developing the algorithm, and its accuracy was evaluated on the independent sample described in the “Sensitivity analyses: validation of scoring system” section, which has not been utilized in any part of the study. The model achieved an accuracy of 79% using only the information from the first visit. The decision tree diagram and the corresponding confusion matrix are shown in Supplementary Fig. S9. The confusion matrix indicates that the model did not misclassify “Cognitive Decliners” as “Positive-Agers” and vice versa.

## Discussion

In this study, we proposed a novel algorithm, OptiCS, designed to distinguish between Positive-Agers and Cognitive Decliners. Initially, the algorithm used cognitive tests to calculate a cognitive score for each individual, subsequently assigning them one of the labels, “Positive-Ager” or “Cognitive Decliner,” through a systematic labeling procedure. Following this, a predictive pipeline was utilized to establish a connection between the features (i.e., dMRI and demographics) and the assigned labels (i.e., cognitive classes). This process was then optimized using an optimization technique based on a constrained adaptive noise scaling algorithm to maximize classification performance.

The contributions of this study can be summarized as follows:

1. Introducing a straightforward and interpretable cognitive scoring function based on FI, PMM, and RT exams to quantify the cognitive performance of individuals;
2. Developing an algorithm to optimally distinguish between cognitive classes using dMRI and demographic data, achieving an AUC of 83%;
3. Uncovering the impact of dMRI attributes and demographic factors on the likelihood of a given participant being classified as Positive-Ager versus Cognitive Decliner;
4. Proposing a predictive framework to identify cognitive classes and estimate the rate of change (i.e., slope) of future cognitive trajectories based solely on demographic information and cognitive exams conducted during the baseline visit.

As mentioned in the “Contribution of features in cognitive classification” section, we found education, age, waist circumference, and sex as the most important demographic predictors (see the “Demographic data” section for the complete set of demographic features) in discriminating between Positive-Agers and Cognitive Decliners. We briefly summarize these findings below.

1. Education: First, we found that higher levels of education were associated with superior cognitive performance in Positive-Agers. This is aligned with previous research studies reporting cognitive reserve in adults with more educational attainment [30–33].
2. Age: We observed a decrease in the positive-aging likelihood as adults age. Aging is often but not always related to subtle progressive decline in cognitive domains like memory, executive function, visuospatial, and language [34, 35].
3. Waist circumference: Previous studies have found conflicting results about the association between waist circumference and cognitive status. In adults aged over 70 years, a larger waist circumference was related to a higher risk of cognitive impairment [36]. Similarly, in both cross-sectional and longitudinal studies among aged non-Hispanic White, Chinese, and African American adults, a larger waist circumference was related to either a greater risk of cognitive impairment or a higher rate of cognitive decline [37–39]. There are conflicting findings, however. For example, in a longitudinal study with four time points over 7 years, there was a lower rate of cognitive decline among Chinese adults aged over 60 years who had increased waist circumference [40]. Similarly, adults aged over 70 years with larger waist circumference showed better performance in memory and processing speed cognitive domains [41]. This picture is further complicated because age modifies how white adipose mass and cognition are related. For instance, among adults aged 60–101 years, a longitudinal study over 8 years found that higher waist circumference was related to worse cognitive impairment in mid-life (40–65 years), but less risk in late-life (i.e., after 65 years). This late-life relationship was stronger in women than men [42].

**Fig. 6:**
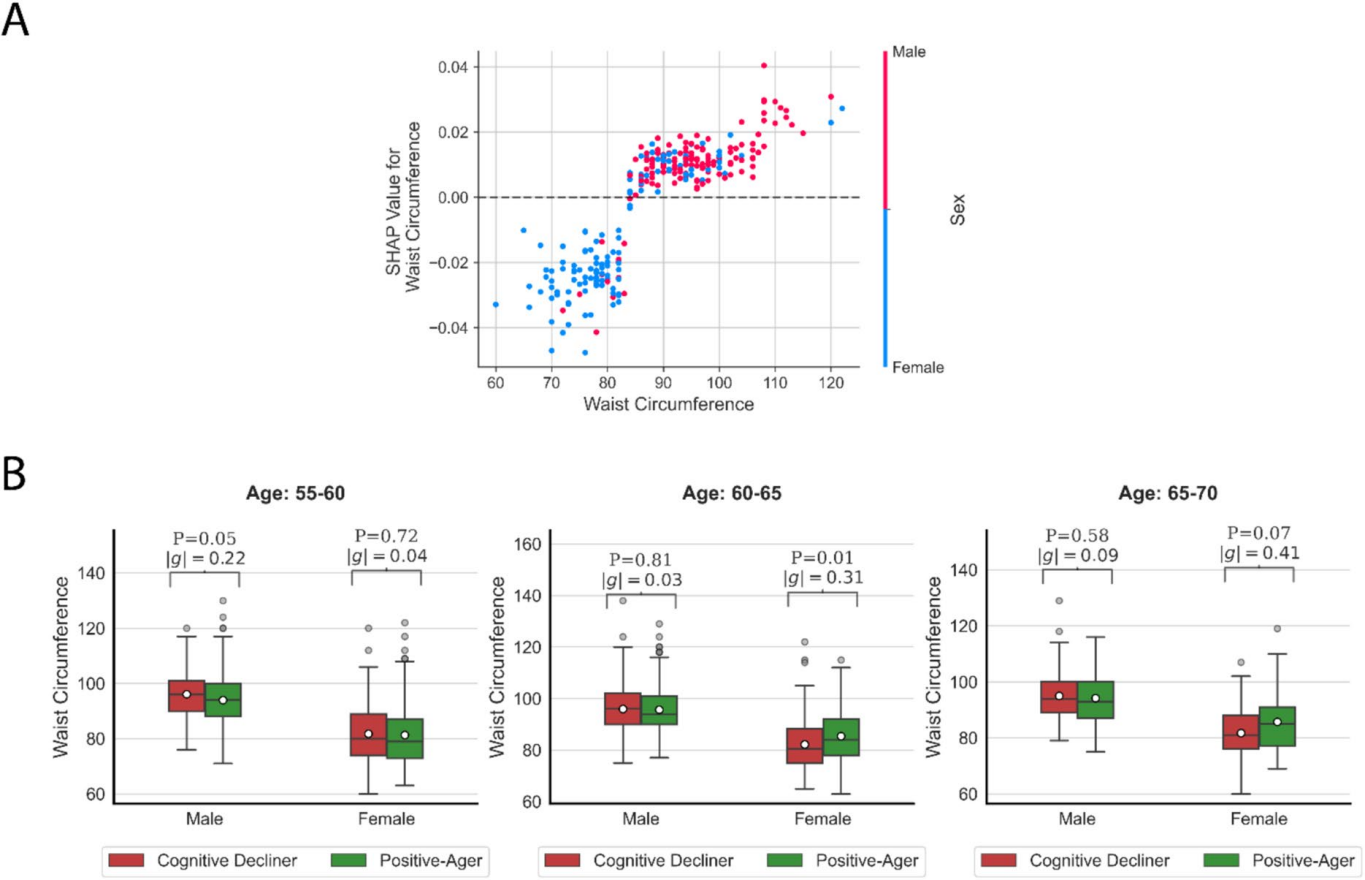
Waist circumference analysis. **A** Dependence plot illustrating the relationship between waist circumference and sex, highlighting their interaction. **B** Boxplot displaying waist circumference values stratified by age groups for each sex. The values of $p$ and $g$ show the *P*-value of the two-sample independent *t*-test and Hedge’s *g* effect size, respectively

Our findings echo these associations. First, stratification by sex turned out to be important, as we identified an interaction effect between sex and waist circumference based on SHAP values (see Fig. 6A). Second, when we analyzed different age groups, we observed a strong and positive correlation between cognition status and waist circumference in older women (see Fig. 6B). There are likewise several studies that revealed a positive association of body mass and cognition in the late-life period, particularly in women [43, 44]. Differences between women and men in abdominal fat associations are associated with hormonal changes across age intervals. Multiple studies reported negative direct and indirect effects of estrogen reduction in cognition [45, 46]. In women who are in their postmenopausal period, estrogen production decreases and remains relatively lower in late life. Because adipose tissues are another source of estrogen production, more abdominal fat compensates for the lack of estrogen in late life which leads to a better cognition status [46, 47].

Here are further summaries for other predictors that significantly loaded:

4. Sex: For sex differences, we found that men were more likely to be in the Positive-Ager group. Previous research has consistently shown that women outperformed men in memory and verbal cognitive exams, while men outperformed women in visuospatial skills [48–50]. Differences in hormones like testosterone play an important role in neural systems and cognition [51].
5. Fornix + stria terminalis: The limbic system is involved in various basic and vital functions like control of emotions, behavior, and memory [52]. The amygdala, hippocampus, thalamus, hypothalamus, basal ganglia, cingulate gyrus, and fornix are the most important components of the limbic system [52]. The hippocampus, which is located deep within the temporal lobe of the cerebral cortex, plays an important role in learning and memory [53]. The fornix is an arched white tract connecting the hippocampus to other brain regions, and it is involved in episodic memory and other cognitive functions [54]. The stria terminalis is a band of white fibers between the caudate nucleus and the thalamus involved in controlling autonomic and behavioral activities [55]. Fibers of stria terminals continue along in the column of the fornix [55]. Adults at high risk of AD and all-cause dementia exhibit lower mean values of MO in the fornix and stria terminalis compared to healthy individuals [56]. Similarly, our study found that mean MO values in the fornix and stria terminalis below 0.45 were associated with a reduction in the likelihood of positive aging by up to 7%. This suggests that microstructural integrity among crossing fibers involved in memory processing is key to maintaining cognitive flexibility associated with a lack of cognitive decline.
6. Mean L1 in medial lemniscus: The medial lemniscus is part of the dorsal column-medial lemniscus pathway. Classically, this pathway carries peripheral vibration, conscious proprioception, fine touch, and two-point discrimination from the extremities and trunk up to the medulla, thalamus, and eventually the primary somatosensory cortex. Although rarely studied in the context of cognition, some studies suggest that damage to the medial lemniscus is related to cognitive deficits or clinical impairment in spinocerebellar ataxia [57], traumatic spinal cord [58] or brain injury [59], multiple sclerosis [60, 61], and stroke [62]. We found that high mean L1 values of the medial lemniscus (larger than 0.0014) were associated with an increase in the likelihood of positive-aging by up to 4%.
7. Mean OD and L3 in cerebral peduncle: The cerebral peduncles include fibers and white matter tracts connecting the cerebellum to other areas of the central nervous system [63]. The cerebellum is a vital part of the hindbrain and coordinates not only movements but also some cognitive functions [64]. Higher OD values in white matter regions, such as the cerebral peduncle, are associated with worse cognitive status due to neural degeneration [65]. Consistent with this, our results indicated that mean OD values exceeding 0.104 in the cerebral peduncle are linked to a reduction in the likelihood of positive aging by up to 2.5%.

Other diffusion measures are also relevant to healthy aging or decline. For example, adults diagnosed with AD and an average age of 70.5 years showed lower FA and higher RD measurement in the medial and superior cerebral peduncle in comparison with healthy elders who were slightly younger (mean of 67.2 years) [66]. We found that higher L3 in the cerebral peduncle was associated with a higher probability of being in the Cognitive Decliner group, which is consistent with the previous findings, as RD is the mean of L2 and L3. Moreover, we found that higher FA values in the cerebral peduncle increase the predicted probability of being in the Positive-Ager group (see Supplementary Fig. S5).

8. Mean L1 in tapetum: Older adults with unimpaired cognition have lower AxD (i.e., less water diffusion and thus more tract integrity of fibers parallel to major tracts) compared to adults with MCI or AD because of axonal degeneration [67]. Our findings support this observation in healthy adults, demonstrating that those with lower mean L1 values in the tapetum are more likely to belong to the Positive-Ager group.
9. Mean L1 in superior cerebellar peduncle: The superior cerebellar peduncle is one of the three paired cerebellar peduncles which connects the cerebellum to the brainstem. Cerebellar peduncles are involved in cognitive functions and mobility. One study found that lower AxD in cerebellar peduncles, including superior, middle, and inferior cerebellar peduncles, was associated with higher age and worse mobility status among cognitively healthy adults [68]. We found that mean L1 values lower than 0.00157 were associated with a decrease in Positive-Aging likelihood by up to 3%.

Besides exploring the relationship between dMRI and demographics with cognitive classes, we introduced a longitudinal cognitive trajectory analysis. The slope trajectory analysis conducted in this study provides several benefits. First, it empowers analysts with a predictive tool for estimating the cognitive trajectory slope of a patient, given the patient’s current age. Second, it establishes a basis for cognitive monitoring over time and evaluates the extent to which an individual’s cognitive score may change in subsequent visits. Third, by comparing the cognitive trend of a given patient with the average trend observed for a specific cognitive class, we can assess where the patient stands relative to the average population.

Finally, the analysis of cognitive scoring and labeling on both in-sample and out-of-sample data demonstrated a clear separation between cognitive classes across various cognitive aspects. As an additional step, we explored the possibility of predicting cognitive class labels using data collected during the first visit (${t}_{1}$), including demographic information and results from the FI, PMM, and RT exams.

## Conclusion

In summary, this study proposes a simple yet effective mathematical definition for cognitive classes (i.e., “Positive-Agers,” “Cognitive Decliners,” and “Normal-Agers”) based on three cognitive tests: fluid intelligence score, pair-matching memory, and reaction time. The extreme cognitive classes of Positive-Agers and Cognitive Decliners were predicted using demographic and dMRI features, achieving an AUC of 83%. This enabled us to quantify the impact of dMRI attributes and demographic data on the likelihood of positive aging.

This study has distinctive strengths. One of its unique aspects is that it is the first cognitive classification study utilizing dMRI attributes. To this end, a novel solution was designed with dual benefits: (I) Given the cognitive test results for a participant, it calculates a cognitive score, which translates into the degree to which someone belongs to a certain cognitive class, and (II) given the dMRI attributes and demographic information, we were able to predict the cognitive classes with high accuracy. Another strength of this study is the exploration of the relationship between dMRI attribute values and cognitive performance. We showed the direction of impact and its magnitude on the likelihood of positive aging. Finally, this study proposed a predictive framework to estimate future cognitive trajectories based on the first visit, which might be a promising tool in the early detection of severe cognitive declines in healthy adults and potential times to apply drug or non-drug therapies.

There are several practical applications of this study in clinical settings. First, OptiCS offers a standardized procedure for quantifying cognitive performance using a set of cognitive exams. The resulting cognitive score can be applied to longitudinal cognitive assessments, providing a reliable measure of an individual’s cognitive performance over time. Given its cost-effectiveness, clinicians could incorporate this as a routine evaluation to monitor and track cognitive health regularly. Second, the proposed mechanism in the “Constructing the average cognitive trajectory curve” section could be utilized to detect potential future cognitive decline by leveraging baseline cognitive test results and demographic data. This serves as a complementary predictive tool, capable of raising an alert when evidence suggests a high risk of future decline. However, to achieve such broad applicability, two key considerations must be addressed: (I) verification of the proposed cognitive scoring system using independent cohorts and (II) appropriate adjustments to the cognitive scoring function to account for varying longitudinal intervals between examinations.

It is also essential to acknowledge the limitations inherent in this study, which pave the way for future research directions. First, validation of the proposed cognitive scoring system on independent cohorts is needed to determine if it is a generalizable solution applicable across different populations. Second, while we used three cognitive measures to create the cognitive scoring system, future research might explore identifying an optimal subset of cognitive tests that can improve the separation between cognitive groups. Third, although XGBoost is an advanced model capable of capturing highly nonlinear relationships, this study did not study the interactions between input features in detail. Further decomposition of the impacts identified in this study into main and interaction effects for the input features could reveal more information about the existing interactions between features. Fourth, this study focused on white matter tracts and demographic information for cognitive classification. However, the goal was not necessarily to develop the most comprehensive model, as several important factors—such as genetics, sleep, emotional and social support, psychological factors, physical activity, and nutrition—were not included but may influence cognitive outcomes. Future research should incorporate these covariates to enhance the model’s comprehensiveness and accuracy.

## Supplementary Information

Below is the link to the electronic supplementary material.

Supplementary file1 (PDF 1336 KB)

## Data Availability

Data is not public and is accessed from UK Biobank. We have referred to UK Biobank in the Data Section and described it in details.
