# Supplementary material for: Decoding cognitive aging: how white matter tracts and demographics distinguish potential Super-Agers
Source: GeroScience. 2025 Feb 26;47(3):4635–61. doi: 10.1007/s11357-025-01566-0 (PMC12181581; doi:10.1007/s11357-025-01566-0)
Supplement: Supplementary file 1 — Supplementary file1 (PDF 1336 KB) [file 11357_2025_1566_MOESM1_ESM.pdf]

1. Supplementary Figures

Supplementary Figure S1. Pseudocode for the OptiCS algorithm

Algorithm: Main Algorithm

Inputs: $X^{train}, X^{val}, D^{train}, D^{val}, J$
Outputs: Θ^*

Initialize Θ
for iteration=1…J **do**

1. Calculate cognitive scores: $\gamma^{train} = f\left(X^{train} \middle| \Theta\right)$.
2. Find lower and upper thresholds:
   $lb = Q_q(\gamma^{train})$ and $ub = Q_{1-q}(\gamma^{train})$.
3. Apply labeling procedure:
   $$y_i = \begin{cases} \text{"Cognitive Decliner"}, & if\ \gamma_i \leq lb \\ \text{"Positive} - \text{Ager"}, & if\ \gamma_i \geq ub \end{cases}, \quad \forall\, i = \{1, 2, \dots, N\}$$
4. Apply the same labeling on the validation set.
5. Find the best predictive pipeline $g\left(D^{train} \middle| \Theta\right)$.
6. Evaluate the predictive pipeline g on the validation set:
   $\mathcal{L}(\Theta) = 1 - AUC\left(g\left(D^{val}|\Theta\right)\right)$
7. Apply the Constrained Adaptive Noise Scaling algorithm to find the next Θ.

end
$\Theta^* = \underset{\Theta}{\operatorname{argmin}}\{\mathcal{L}(\Theta^{(1)}), \mathcal{L}(\Theta^{(2)}), \dots, \mathcal{L}(\Theta^{(J)})\}$

Supplementary Figure S2. Bar plot of the aggregated importance of various brain regions based on the SHAP values. The height of each bar is the sum of mean absolute SHAP values for all dMRI attributes corresponding to a particular tract, peduncle, or other white matter structure.

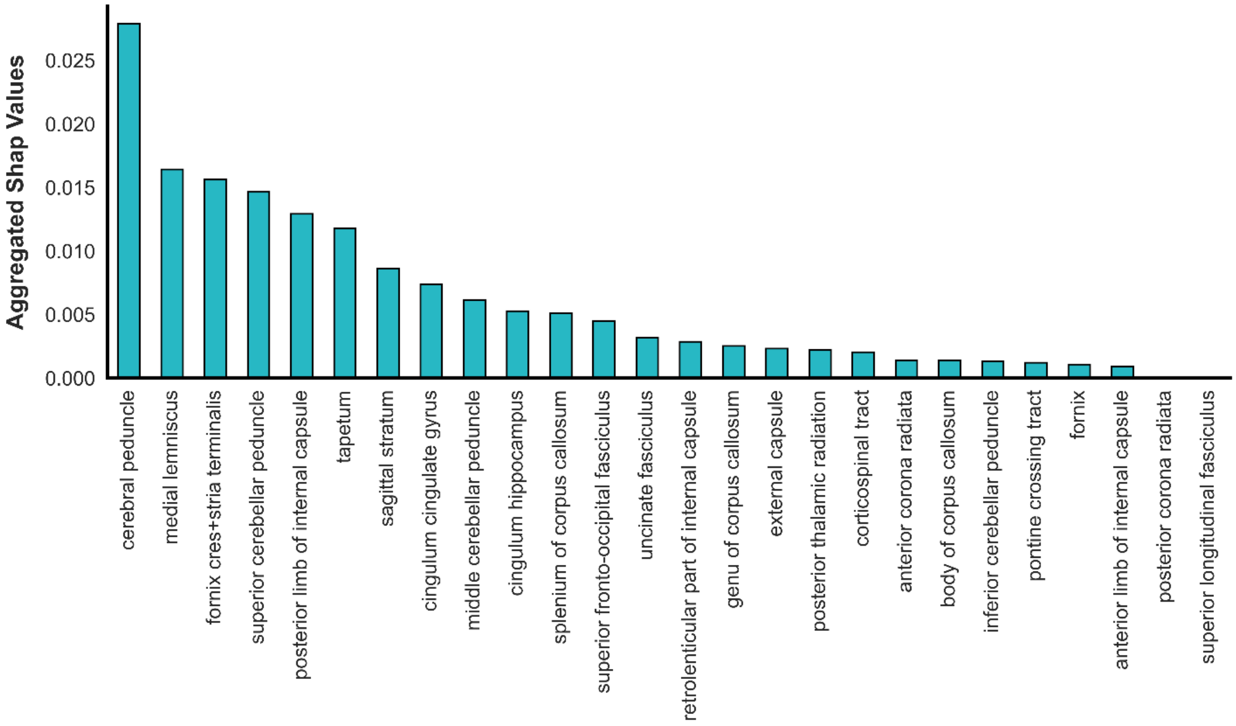

Supplementary Figure S3. Difference between Positive-Agers and Cognitive Decliners in top demographics features that loaded into the final model. A) Bar plots of Other Education, Secondary Education, or Post-Secondary/Vocational education levels; B) Box plot and estimated kernel density plot of age in years; C) Box plot and kernel density plot of waist circumference; D) Bar plot of the proportion of cognitive classes in males and females.

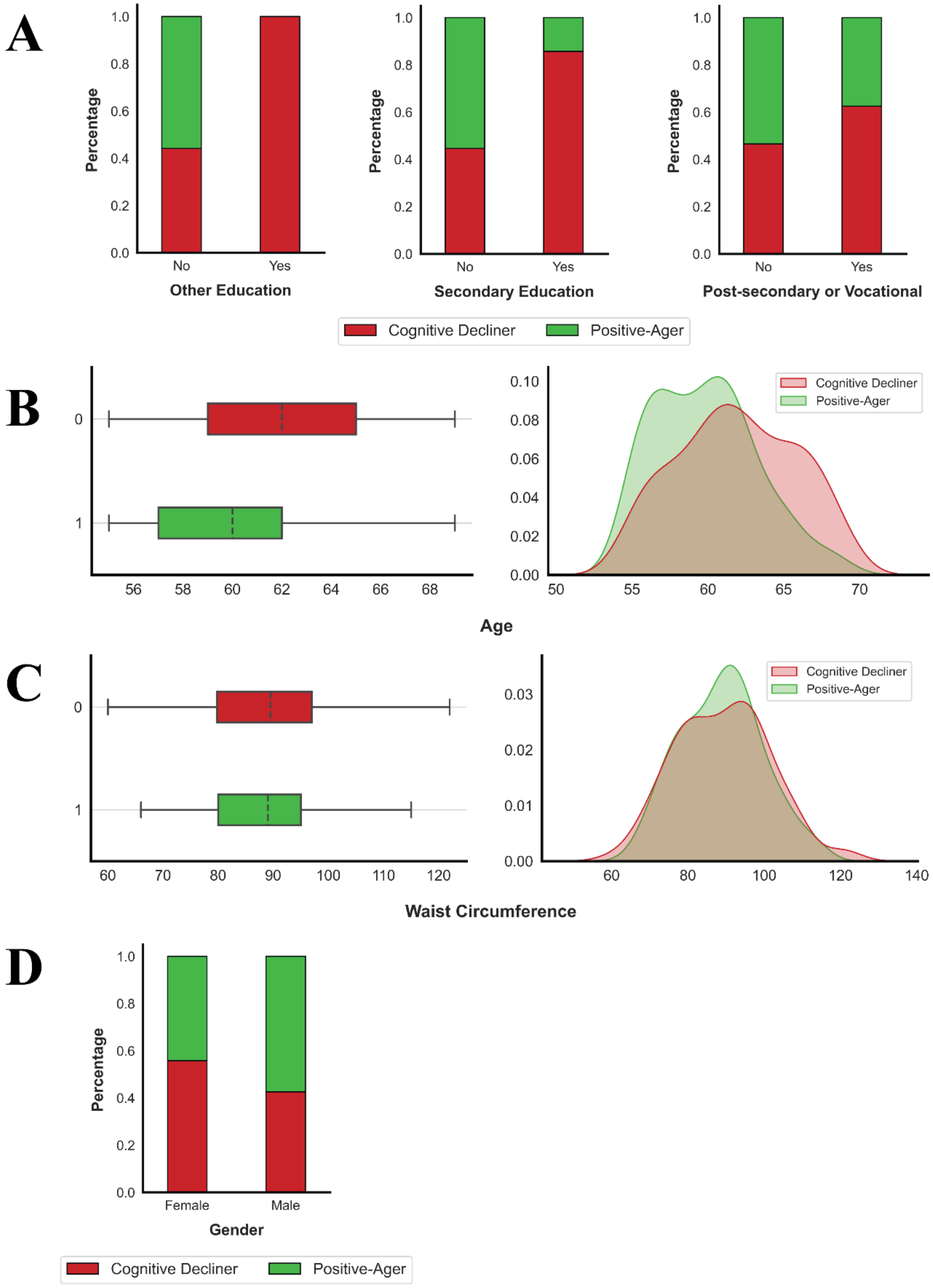

Supplementary Figure S4. Box plots of the remaining dMRI features (from 7th to 24th most important) for the two cognitive classes “Cognitive Decliner” and “Positive-Ager”. This supplementary figure is complementary to Figure 4A in the main text.

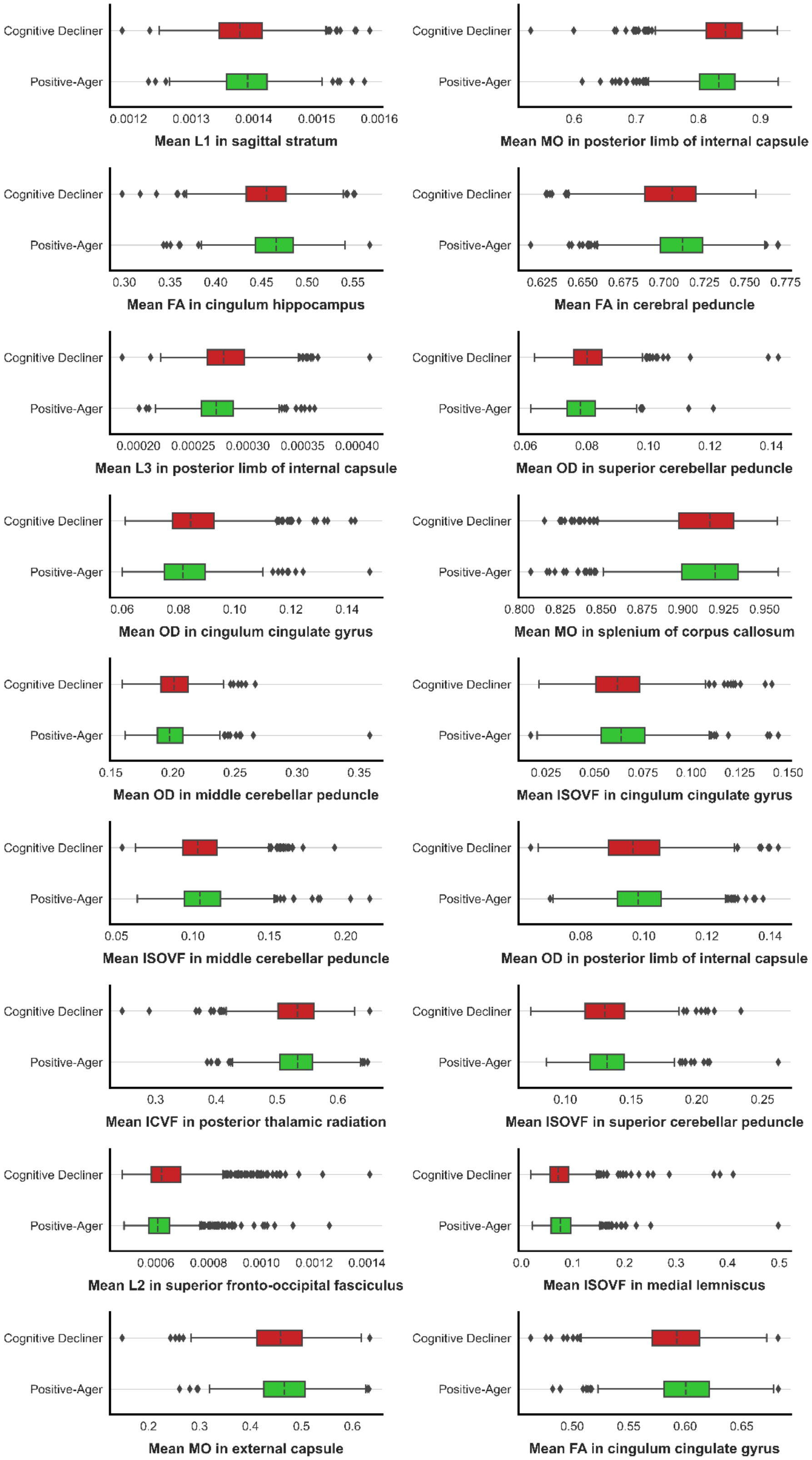

Supplementary Figure S5. Effect of the remaining dMRI features (7th to 24th most important dMRI attributes) on Positive-Aging likelihood. The Y-axis shows SHAP values for a given feature: a higher value on the Y-axis corresponds to a greater impact of a given feature on the predicted probability of the Positive-Aging class. The X-axis represents values for a given type of diffusion metric (e.g., L1, L2, ISOVF, etc.). Each subplot has a title with the corresponding feature and its rank. The rank of a feature represents the position of the feature compared to all existing variables according to the feature importance. This supplementary figure is complementary to Figure 4B in the main text.

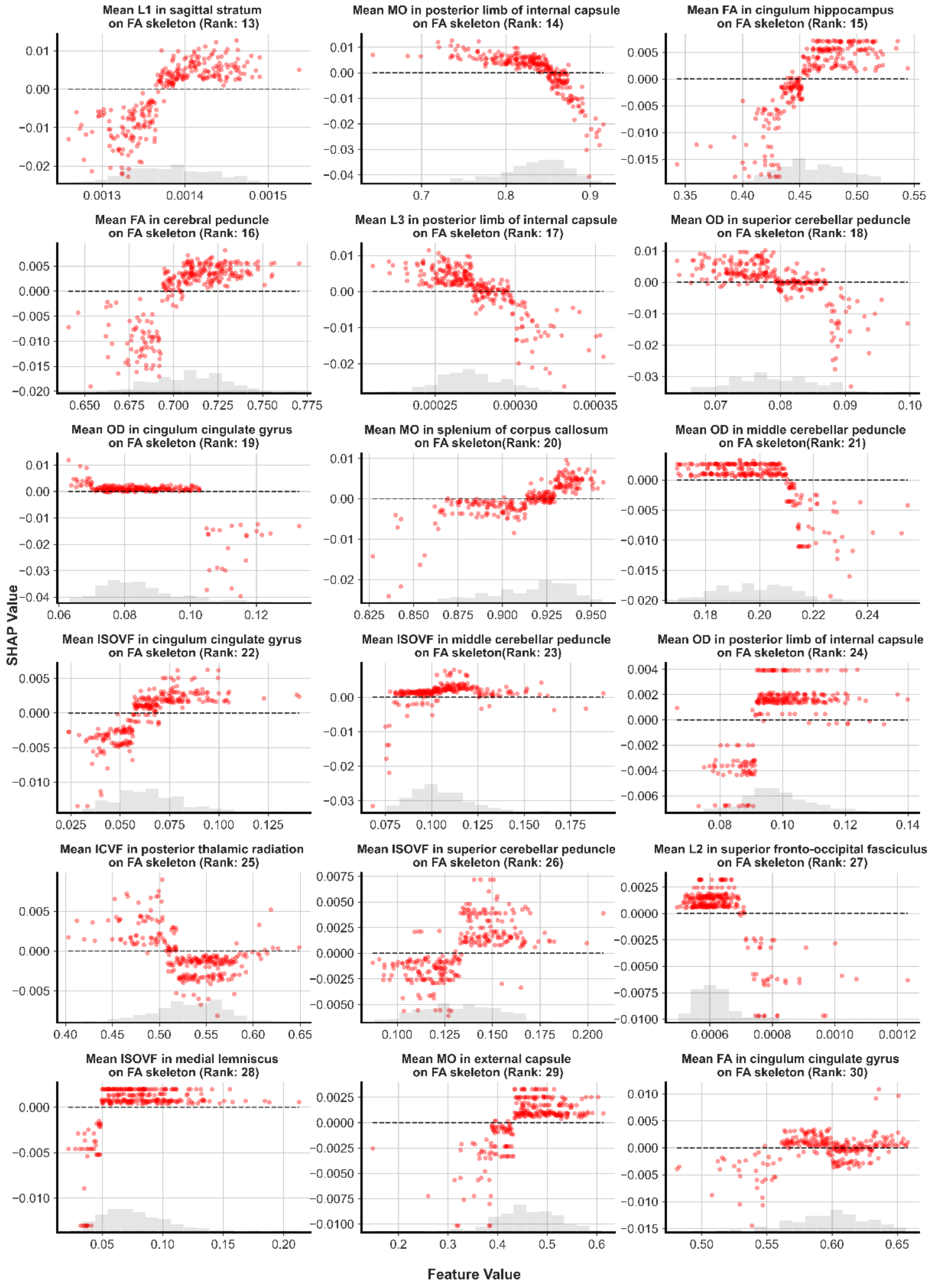

Supplementary Figure S6. Optimal line equations separating cognitive classes.

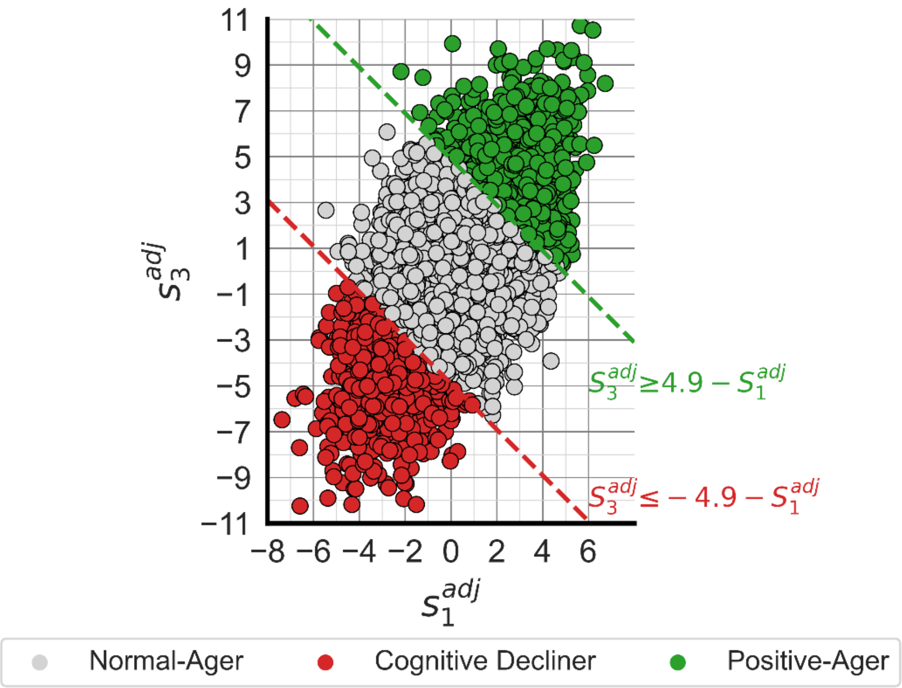

Supplementary Figure S7. Box plots of trajectory slopes computed using different methods. We compared four different approaches for computing the slope (i.e., rate of change) between time points t_1 and t_3: I) Individual FI, PMM, and RT exams; II) Simple average of all cognitive exams (i.e., equal weights); III) Weighted average of cognitive tests using the PCA loading vector; IV) Weighted average of cognitive tests using optimal weights (equivalently, S_1^{adj} and S_3^{adj}). The values on top of the box plot show the absolute value of Hedge's g effect size. Both visual and statistical methods confirm that optimal weights create a better separation between cognitive exams.

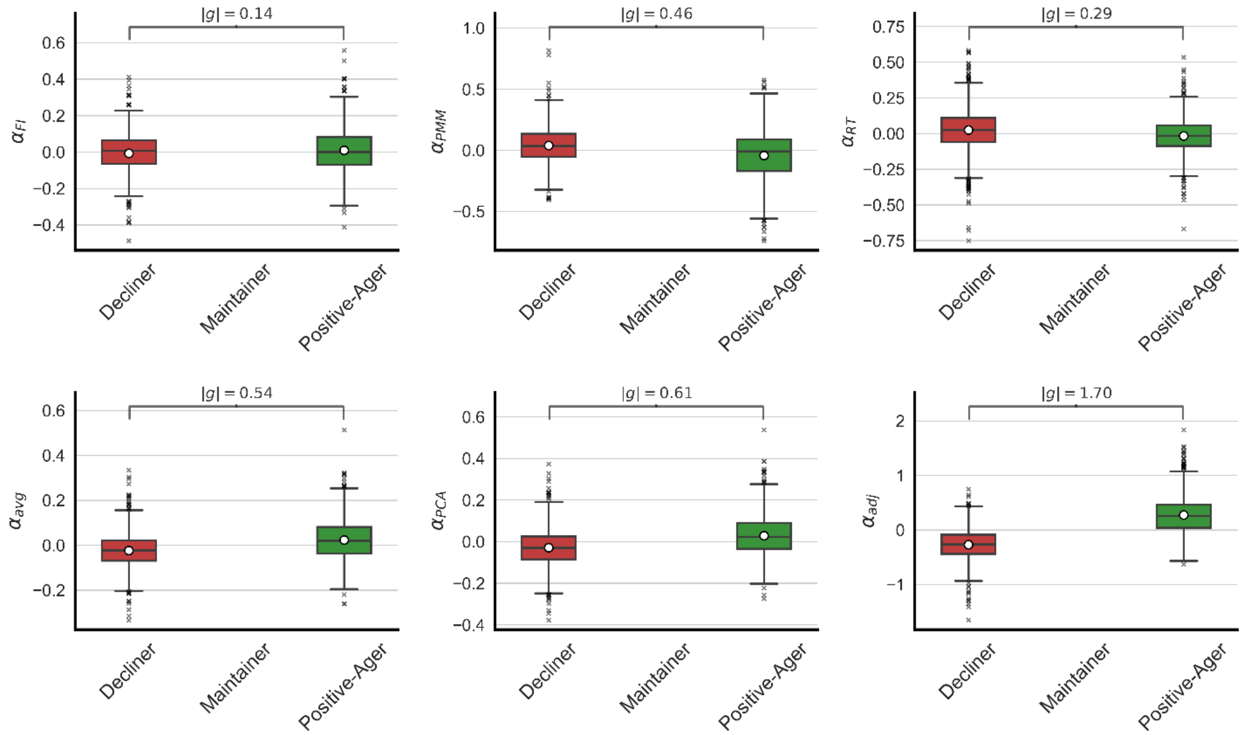

Supplementary Figure S8. Relation between cognitive scores (γ) and adjusted trajectory slope (α_{adj}). There is a positive correlation between the calculated cognitive score and trajectory slope ($\rho = 0.45$). Individuals with very high cognitive scores close to one (i.e., Positive-Agers), shown in green, are more likely to have positive slopes, indicating improvement over time. Conversely, those with very low cognitive scores close to zero (i.e., Cognitive Decliners), shown in red, tend to have smaller and more negative slopes, indicating a decline in their cognitive performance. Similarly, a positive correlation between cognitive score and trajectory slope is observed within each cognitive group. Higher scores in Positive-Agers and smaller values of cognitive scores in Cognitive Decliners were associated with larger improvement ($\rho = 0.30$) and steeper decline ($\rho = 0.22$) in cognitive performance, respectively.

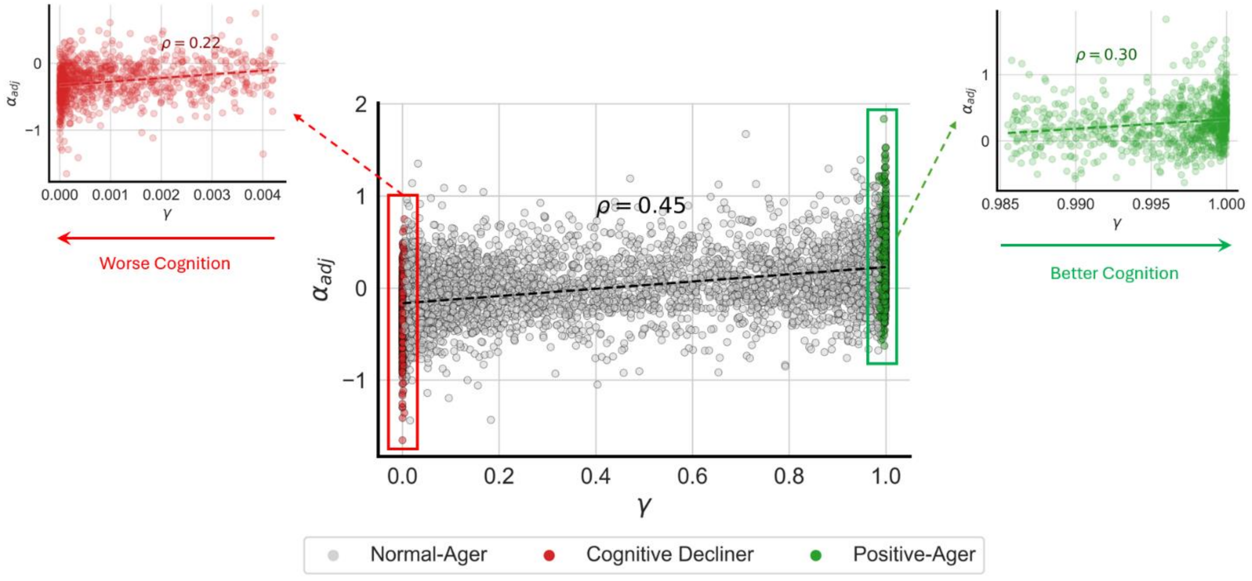

Supplementary Figure S9. Decision tree model. A) Decision tree output for predicting the cognitive class using only baseline visit information. Green, orange, and purple nodes show Positive-Agers, Normal-Agers, and Cognitive Decliners, respectively. The model only used the adjusted score at baseline (S_1^{adj}), age at baseline (t_1), household income level, income, and BMI. B) Confusion matrix of the decision tree model using an independent test dataset.

A

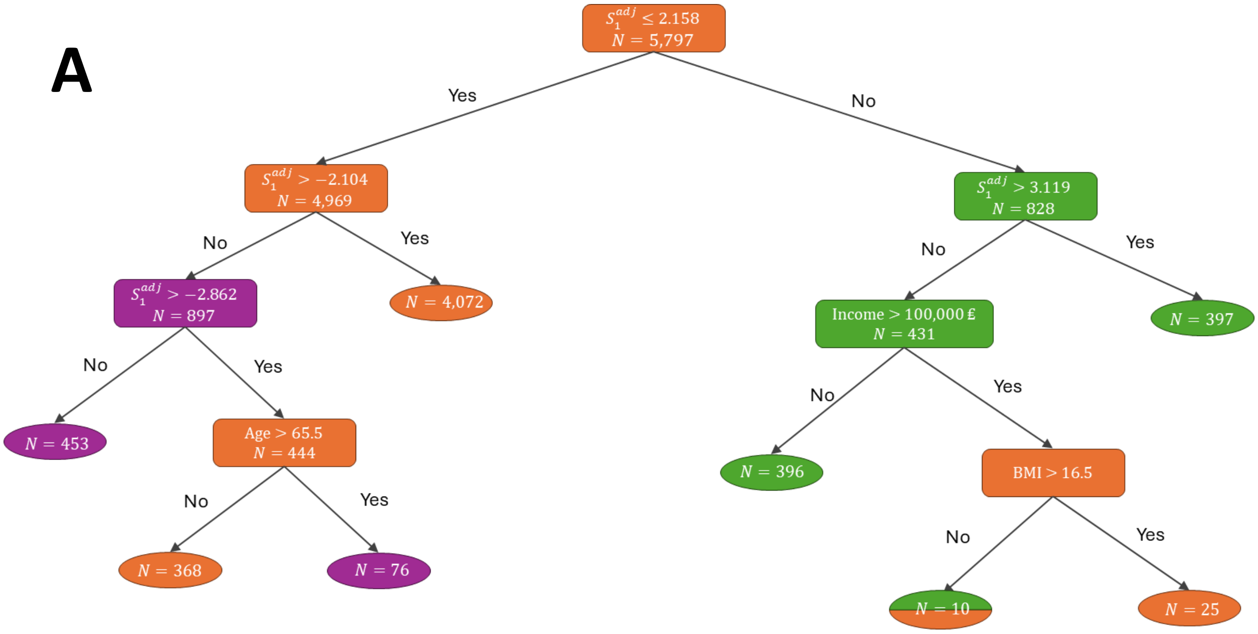

B

		Predicted			Class Sensitivity
		Cognitive Decliner	Normal-Ager	Positive-Ager	
Labels	Cognitive Decliner	137	34	0	80%
	Normal-Ager	119	688	216	67%
	Positive-Ager	0	31	257	89%

2. Supplementary Tables

Supplementary Table S1. Summary of scoring function's parameter description and feasible domain.

Parameter	Corresponding Exam	Feasible Domain	Initial Value
$\boldsymbol{\theta_0}$	Bias (intercept)	$\mathbb{R}$	0
$\boldsymbol{\theta_1}$	FI_1	$\mathbb{R}_{\geq 0}$	1
$\boldsymbol{\theta_2}$	FI_3	$\mathbb{R}_{\geq 0}$	1
$\boldsymbol{\theta_3}$	PMM_1	$\mathbb{R}_{\leq 0}$	-1
$\boldsymbol{\theta_4}$	PMM_3	$\mathbb{R}_{\leq 0}$	-1
$\boldsymbol{\theta_5}$	RT_1	$\mathbb{R}_{\leq 0}$	-1
$\boldsymbol{\theta_6}$	RT_3	$\mathbb{R}_{\leq 0}$	-1

Supplementary Table S2. Values selected for the hyperparameters of the algorithm.

Hyperparameter	Value	Description	Component
$\boldsymbol{q}$	{0.10, 0.15, 0.20, 0.25}	Quantile threshold	Labeling
$\boldsymbol{m_{fs}}$	50	Maximum depth of decision tree	Prediction
$\boldsymbol{\mathcal{F}}$	150	Reduced feature space size	Prediction
$\boldsymbol{J}$	50	Number of iterations	Optimization
$\boldsymbol{NC}$	5	Number of candidate solutions	Optimization
$\boldsymbol{\sigma}$	0.4	Initial noise scale	Optimization
$\boldsymbol{\sigma_u}$	1.2	Noise upscaling coefficient	Optimization
$\boldsymbol{\sigma_l}$	0.8	Noise downscaling coefficient	Optimization
$\boldsymbol{\sigma_{max}}$	5.0	Maximum noise scale	Optimization

Supplementary Table S3. Description of hyperparameters in G. The "Range/Values" column shows the search space explored during the tuning (optimization) process and the "Best Value" column represents the optimal values found and used in this study.

Hyperparameter	Description	Range/Values	Best Value
$\boldsymbol{r_{col}}$	Subsample ratio of columns	(0.1, 1)	0.91
$\boldsymbol{\zeta}$	Minimum required loss reduction	(0, 5)	3.32
$\boldsymbol{\eta}$	Learning rate	(0.01, 0.3)	0.05
$\boldsymbol{m_G}$	Maximum depth of trees in XGBoost	{3, 4, …, 50}	11
$\boldsymbol{w_c}$	Minimum required sum of instance weight	[1, 10]	1
$\boldsymbol{\mathcal{N}_G}$	Number of trees in XGBoost	{50, …, 500}	116
$\boldsymbol{r_{ins}}$	Subsample ratio of the training instances	[0.1, 1]	0.89
$\boldsymbol{m_{fs}}$	Maximum depth of tree in feature selection	{20, …, 100}	29
$\boldsymbol{\mathcal{F}}$	Reduced feature space size in feature selection	{40, …, 150}	88
$\boldsymbol{M_{BO}}$	Number of iterations in Bayesian Optimization	40	-

Supplementary Table S4. Sex dimorphism. Descriptive statistics for demographic features stratified by sex.

		Cognitive Decliner		Positive-Ager	
		**Female (471)**	**Male (408)**	**Female (342)**	**Male (522)**
**Age**	-	61.2 ± 4.0	62.2 ± 3.9	59.6 ± 3.5	60.1 ± 3.4
**BMI**	-	17.5 ± 2.9	18.6 ± 2.5	18.2 ± 3.1	18.6 ± 2.7
**Waist Circumference**	-	81.9 ± 9.8	95.7 ± 8.9	83.2 ± 10.9	94.7 ± 9.7
**Household Income**	Lower class	33.1%[a]	32.8%	24.6%	19.3%
	Middle class	28.7%	25.7%	29.5%	32.8%
	Under class	23.6%	21.8%	10.8%	7.3%
	Upper class	2.8%	3.2%	6.1%	9%
	Upper-Middle class	11.9%	16.4%	28.9%	31.6%
**Handedness**	Ambidextrous	0.6%	3.4%	0.9%	1%
	Left-Handed	9.6%	11.5%	5.8%	10%
	Right-Handed	89.8%	85%	93.3v	89.1%
**Education**	College or similar	51%	49.8%	83.3v	84.7%
	Other education	14%	17.9%	0.3%	0.2%
	Post-secondary or Vocational	14.4%	23%	10.2%	11.9%
	Secondary education	20.6%	9.3%	6.1%	3.3%
**Skin Color**	Brown-Skinned	0%	1%	0%	0.2%
	Olive-Skinned	21.9%	20.8%	18.7%	17.6%
	Pale-Skinned	78.1%	78.2%	81.3%	82.2%
**Tobacco Use**	Non-smoker	64.5%	54.2%	64%	58.8%
	Prior smoker	31.6%	41.9%	33%	36.2%
	Smoker	3.8%	3.9%	2.9%	5%
**Tobacco Type**	Cigars Pipes	0.2%	0.5%	0%	1%
	Hand-rolled Cigs	0%	1%	0%	0.6%
	Manufactured Cigs	2.8%	1.7%	0.6%	2.1%
	Non-smoker	97%	96.8%	99.4%	96.4%

[a] Percentage is calculated within each sex and cognitive group.

3. Supplementary Text

A. Why not rely on a 'Demographics Only' model to predict the cognitive classes?

First, while demographic data alone can perform reasonably, there is still a notable gap between its performance and that of the combined feature set. As an example, for q=0.15, the 'Demographics Only' model achieves the same accuracy (72%) and even better recall (75%). However, there is a 7% drop in the AUC and a 6% reduction in specificity when ignoring dMRI. Second, looking at the SHAP values, the sum of the average impact on Positive-Aging likelihood computed for demographic data was 0.18. Comparing this value with that of dMRI data, 0.16, we noticed a small difference of 0.02. This difference indicates that dMRI attributes are: 1) equally important; and 2) the results are not influenced solely by demographic features when using both sets of features (see **Section 3.2** in the main text). Third, the specificity of the "Demographics Only" model is low, which means those who were truly "Cognitive Decliners" were not correctly classified. This would raise some concerns in clinical settings. Finally, beyond improvements in performance metrics, the inclusion of dMRI features allows for an exploration of the relationship between cognitive performance and dMRI attributes. This helps in understanding the underlying biological mechanisms and their association with cognitive performance, providing valuable insights that would be missed if demographic data were only used.

B. Definition of cognitive tests in the independent sample

Digit Symbol Substitution Test (**DSST**): This neuropsychological test focuses on associative learning. First, there is a key at the top of the page, indicating specific symbols that represent specific numbers (e.g., 'ε' represents '8'). There are then boxes aligned in rows and columns, with each box having a number. The objective is to correctly substitute a given symbol with a given number. Classically, for example, the boxes '2', '9', and '2' would require writing in the following symbols: '↨', 'э', '↨'.

Number Memory Test: This is a neuropsychological test that probes short-term memory. In the standard "forward" format, the goal is to remember a number of digits presented in the same sequence, with the number of digits increasing by one for each correct iteration. For example, a 3-digit span might require viewing the sequence 946 and selecting 9, 4, and 6. This would be followed by a 4-digit span and so on until a mistake is made. This is denoted as the "Max Digits" test in our study.

Trail Making Test, Parts A and B (**"Trails A and B"**): "Trails A" is an attention-based neuropsychological test. The goal is to connect the numbers 1 to 25 on a piece of paper, where the numbers are in arbitrary positions on the paper. "Trails B" is more difficult and requires both attention and task-switching cognitive processes. Here, the goal is to alternate between thirteen numbers (1 to 13) and twelve letters (A to L). The correct pattern would be A-1-B-2-C-3, and so on.

C. Average Cognitive trajectory curves for cognitive classes

Below, we listed the average cognitive trajectory curves for Positive-Agers, Cognitive Decliners, and Normal-Agers, where the baseline age is from 55 to 70 years old (i.e., $55 \leq t \leq 70$):

$$\mathcal{C}_{positve} = -0.00085\, t^2 + 0.3735\, t + 2.69 \tag{C1}$$

$$\mathcal{C}_{normal} = -0.0026\, t^2 + 0.326\, t + 0.05 \tag{C2}$$

$$\mathcal{C}_{decline} = -0.0022\, t^2 + 0.002\, t - 2.8 \tag{C3}$$
